# Supplementary material for: Association of lipocalin-2 and low-density lipoprotein receptor-related protein-1 (LRP1) with biomarkers of environmental enteric dysfunction (EED) among under 2 children in Bangladesh: results from a community-based intervention study
Source: BMJ Paediatr Open. 2021 Aug 4;5(1):e001138. doi: 10.1136/bmjpo-2021-001138 (PMC8340289; doi:10.1136/bmjpo-2021-001138)
Supplement: Supplementary data [file bmjpo-2021-001138supp001.pdf]

Exact p- value for "**Table 3** Correlation of LCN 2 and LRP1 with others fecal biomarkers and EED score"

A significant positive correlation was found among LCN2 and different biomarkers of intestinal inflammation like MPO (r = 0.19, **p = 0.005**), NEO (r = 0.20, **p = 0.004**), Calprotectin (r= 0.3, **p = 0.0001**), and Reg1B (r = 0.20, **p = 0.003**). LCN2 also positively correlated with EED score (r = 0.20, **p = 0.003**). On the other hand, LRP1 was negatively correlated with MPO (r = - 0.18, **p = 0.006**), NEO (r = -0.30, **p = 0.0001**), A1AT (r = - 0.18**, p = 0.006**), Reg1B (r= -0.2, **p = 0.003**) as well as EED score (r = -0.29, **p = 0.0001**).
