# Supplementary material for: Association of lipocalin-2 and low-density lipoprotein receptor-related protein-1 (LRP1) with biomarkers of environmental enteric dysfunction (EED) among under 2 children in Bangladesh: results from a community-based intervention study
Source: BMJ Paediatr Open. 2021 Aug 4;5(1):e001138. doi: 10.1136/bmjpo-2021-001138 (PMC8340289; doi:10.1136/bmjpo-2021-001138)
Supplement: Author's manuscript [file bmjpo-2021-001138.draft_revisions.pdf]

BMJ Paediatrics Open

BMJ Paediatrics Open is committed to open peer review. As part of this commitment we make the peer review history of every article we publish publicly available.

When an article is published we post the peer reviewers' comments and the authors' responses online. We also post the versions of the paper that were used during peer review. These are the versions that the peer review comments apply to.

The versions of the paper that follow are the versions that were submitted during the peer review process. They are not the versions of record or the final published versions. They should not be cited or distributed as the published version of this manuscript.

BMJ Paediatrics Open is an open access journal and the full, final, typeset and author-corrected version of record of the manuscript is available on our site with no access controls, subscription charges or pay-per-view fees (http://bmjpaedsopen.bmj.com).

If you have any questions on BMJ Paediatrics Open's open peer review process please email info.bmjpo@bmj.com

BMJ Paediatrics Open

Association of Lipocalin-2 and Low-density lipoprotein receptor related protein-1 (LRP1) with biomarkers of Environmental Enteric Dysfunction (EED) among under 2 children in Bangladesh: Results from a community based intervention study

Journal:	*BMJ Paediatrics Open*
Manuscript ID	bmjpo-2021-001138
Article Type:	Original research
Date Submitted by the Author:	20-Apr-2021

Complete List of Authors:	Hasan, Md. Mehedi ; International Centre for Diarrhoeal Disease Research Bangladesh<br>Gazi, Md Amran; International Centre for Diarrhoeal Disease Research Bangladesh, NCSD<br>Das, Subhasish; International Centre for Diarrhoeal Disease Research Bangladesh<br>Fahim, Shah Mohammad ; International Centre for Diarrhoeal Disease Research Bangladesh<br>Hossaini, Farzana ; International Centre for Diarrhoeal Disease Research Bangladesh<br>Alam, Md. Ashraful ; International Centre for Diarrhoeal Disease Research Bangladesh,<br>Mahfuz, Mustafa; International Centre for Diarrhoeal Disease Research Bangladesh<br>Ahmed, Tahmeed; International Centre for Diarrhoeal Disease Research, Bangladesh (icddr,b), Clinical Sciences Division (CSD); Centre for Nutrition and Food Security (CNFS)

Keywords:	Biochemistry, Gastroenterology, Growth

SCHOLARONE™
Manuscripts

I, the Submitting Author has the right to grant and does grant on behalf of all authors of the Work (as defined in the below author licence), an exclusive licence and/or a non-exclusive licence for contributions from authors who are: i) UK Crown employees; ii) where BMJ has agreed a CC-BY licence shall apply, and/or iii) in accordance with the terms applicable for US Federal Government officers or employees acting as part of their official duties; on a worldwide, perpetual, irrevocable, royalty-free basis to BMJ Publishing Group Ltd ("BMJ") its licensees and where the relevant Journal is co-owned by BMJ to the co-owners of the Journal, to publish the Work in this journal and any other BMJ products and to exploit all rights, as set out in our licence.

The Submitting Author accepts and understands that any supply made under these terms is made by BMJ to the Submitting Author unless you are acting as an employee on behalf of your employer or a postgraduate student of an affiliated institution which is paying any applicable article publishing charge ("APC") for Open Access articles. Where the Submitting Author wishes to make the Work available on an Open Access basis (and intends to pay the relevant APC), the terms of reuse of such Open Access shall be governed by a Creative Commons licence – details of these licences and which Creative Commons licence will apply to this Work are set out in our licence referred to above.

Other than as permitted in any relevant BMJ Author's Self Archiving Policies, I confirm this Work has not been accepted for publication elsewhere, is not being considered for publication elsewhere and does not duplicate material already published. I confirm all authors consent to publication of this Work and authorise the granting of this licence.

Confidential: For Review Only

Association of Lipocalin-2 and Low-density lipoprotein receptor related protein-1 (LRP1) with biomarkers of Environmental Enteric Dysfunction (EED) among under 2 children in Bangladesh: Results from a community based intervention study

Md. Mehedi Hasan[1], Md. Amran Gazi[1]*, Subhasish Das[1], Shah Mohammad Fahim[1], Farzana Hossaini[1], Md. Ashraful Alam [1], Mustafa Mahfuz[1], Tahmeed Ahmed[1]

[1]Nutrition and Clinical Services Division, International Centre for Diarrheal Disease Research, Bangladesh (icddr,b), Dhaka, Bangladesh

Authors' addresses:

1. Md. Mehedi Hasan, MSc , Senior Research Officer, Nutrition and Clinical Services Division, icddr,b, Dhaka, Bangladesh, Email: md.hasan@icddrb.org
2. Md. Amran Gazi, MSc, Assistant Scientist, Nutrition and Clinical Services Division, icddr,b, Dhaka, Bangladesh, Email: amran.gazi@icddrb.org
3. Subhasish Das, MBBS, MPH, Assistant Scientist, Nutrition and Clinical Services Division, icddr,b, Dhaka, Bangladesh, Email: subhasish.das@icddrb.org
4. Shah Mohammad Fahim, MBBS, MPH; Research Investigator, Nutrition and Clinical Services Division, icddr,b, Dhaka, Bangladesh; Email: mohammad.fahim@icddrb.org
5. Farzana Hossaini, MSc, Senior Research Assistant, Nutrition and Clinical Services Division, icddr,b, Dhaka, Bangladesh, Email: farzanasacky@gmail.com
6. Md. Ashraful Alam, MSc, Assistant Scientist, Nutrition and Clinical Services Division, icddr,b, Dhaka, Bangladesh, Email: mashraful@icddrb.org
7. Mustafa Mahfuz, MBBS, MPH, Associate Scientist, Nutrition and Clinical Services Division, icddr,b, Dhaka, Bangladesh, Email: mustafa@icddrb.org
8. Tahmeed Ahmed, MBBS, PhD, Executive Director, icddr,b, Dhaka, Bangladesh, Email: tahmeed@icddrb.org

***Correspondence to: Md. Amran Gazi,** Nutrition and Clinical Services Division, International Centre for Diarrhoeal Disease Research (icddr,b), Dhaka-1212, Bangladesh, **Email:** amran.gazi@icddrb.org

Word count: Abstract: 266; Main text: 2499

Number of table: 4

Confidential: For Review Only

ABSTRACT

Background Environmental enteric dysfunction (EED) is thought to occur from persistent intestinal inflammation. Studies also revealed the association of Lipocalin-2 (LCN2) and Low-density lipoprotein receptor related protein-1 (LRP1) with intestinal inflammation. Therefore, we intended to explore the relationship of LCN2 and LRP1 with gut inflammation and biomarkers of EED in Bangladeshi malnourished children.

Methods A total of 222 children (Length-for-age z-score (LAZ) <−1) aged 12-18 months were enrolled and provided nutrition intervention for 90 days. The LAZ for 104 participants improved after nutrition intervention while in 118 participants did not improve. Plasma and fecal biomarkers were measured using ELISA. Spearman's rank correlation was done to see the correlation among LCN2, LRP1 and biological biomarkers. Multivariable logistic regression was performed to explore the associated factors of non-improvement of study participants.

Results LCN2 correlates positively with Myeloperoxidase ($r = 0.19$, $p = 0.005$), Neopterin ($r = 0.20$, $p = 0.004$), Calprotectin ($r= 0.3$, $p = 0.0001$), Reg1B ($r = 0.20$, $p = 0.003$) and EED score ($r = 0.20$, $p = 0.003$). Whereas, LRP1 correlates negatively with Myeloperoxidase ($r = - 0.18$, $p = 0.006$), Neopterin ($r = - 0.30$, $p = 0.0001$), Alpha-1-antitrypsin ($r = - 0.18$, $p = 0.006$), Reg1B ($r= -0.2$, $p = 0.003$) and EED score ($r = - 0.29$, $p = 0.0001$). Multivariable logistic regression revealed that lower level of LCN2, ferritin and lower family income were positively associated with the non-improvement of participants.

Conclusions Our findings imply that LCN2 might be a promising biomarker to predict gut inflammation and EED. Whereas, increased level of LRP1 may contribute to alleviating intestinal inflammation.

Key words: Lipocalin-2, LRP1, gut inflammation, EED, Bangladesh

Confidential: For Review Only

What is known about the subject?

- Environmental enteric dysfunction (EED) is highly prevalent in developing countries which affects approximately 40% of all children with age less than 5 years.
- EED causes linear growth faltering or stunting which has the manifestations including reduced neurodevelopmental and cognitive function and increased morbidity and mortality in childhood.
- The main challenge to tackle the EED is lack of non-invasive biomarker signature to diagnose the EED and take proper treatment initiative.

What this study add?

- There are significant positive correlation of Lipocalin-2 and significant negative correlation of LRP-1 with biomarkers of EED.
- Study findings strongly infer that LCN2 might be a promising biomarker to predict gut inflammation and EED
- Study findings also imply that LRP1 could be a therapeutic target to alleviate intestinal inflammation.

Confidential: For Review Only

Confidential: For Review Only

INTRODUCTION

Environmental enteric dysfunction (EED) is a subacute intestinal inflammation highly prevalent in developing countries which is defined by crypt hyperplasia, villous atrophy, reduction of mucus layer and infiltration of lymphocytes in lamina propria [1]. EED affects approximately 40% of all children with age less than 5 years in resource poor settings and causes linear growth faltering or stunting [2]. It is thought to occur from repeated enteric infections due to poor hygiene and unsanitary environmental conditions, which in turn results in intestinal inflammation, a major driver of occurring EED [3]. Studies have elicited the relation of Lipocalin-2 (LCN2) and low-density lipoprotein receptor-related protein-1(LRP1) with intestinal inflammation. LRP1 knockout has been found to be associated with increased intestinal inflammation in animal model [4], and LCN2-deficient mice are more prone to bacterial infection [5]. On the other hand, higher expression of LCN2 has been reported in various inflammatory conditions [6]. Hence, it is necessary to explore the relation of LCN2 and LRP1 with intestinal inflammation in the context of EED.

LCN2 is a bacteriostatic peptide, a member of lipocalin super family, known as neutrophil gelatinase B-associated lipocalin (NGAL) [7 8]. One of the most recognized functions of LCN2 is to prevent the bacterial uptake of iron crucial for their growth [9 10]. It has been reported that LCN2-deficient mice shows higher mortality rates following infection with *Escherichia coli* than wild type [5]. Study also reported that LCN2 shows preventive mechanism against intestinal inflammation through bacterial clearance by phagocytosis in macrophages [7]. Moreover, it exerts defensive mechanism against *Salmonella typhimurium, Klebsiella pneumoniae* and *E. coli* in different studies [11-13]. Besides the defensive mechanism, LCN2 has emerged as promising biomarker of inflammation. It has been reported as hallmark for the onset of kidney diseases as well as indicator of inflammation in various diseases [6 14 15]. However, there is no study that has conducted to investigate the level of LCN2 in Bangladeshi malnourished children. Therefore, the relation of LCN2 with intestinal inflammation and EED are yet to be elucidated.

LRP1 is an endocytic receptor that facilitates the binding and endocytic transportation of a broad range of biological ligands as well as various molecules function in immune system [16-18]. It has a vital role in phagocytosis which is an essential component of the innate immune response, crucial for removal of infectious agents [16 19]. Moreover, LRP1 promotes tissue-resident macrophage-survival by activating Akt pathway [20]. Macrophages show the initial defensive mechanism by eliminate noxious agents including bacteria, irritants and necrotic cells [21], whereas LRP1 deficiency causes increased macrophage apoptosis [22]. LRP1 also mediates

pathogen elimination by scavenging membrane lipoproteins of microorganism during infection [23]. Therefore, it can be hypothesized that LRP1 may reduce the gut inflammation by showing defensive mechanism against microbial invasion. In this study, we intended to investigate the level of LCN2 and LRP1 in malnourished children of Bangladesh and their relations with fecal markers of intestinal inflammation and EED.

METHODS

Study participants, nutritional intervention, study site and ethical consideration

To perform the analysis for this study, data were taken from "Bangladesh Environmental Enteric Dysfunction (BEED)" study. The study protocol was published earlier [24]. It is a community based intervention study to validate noninvasive biomarkers of EED. The intervention package included an egg, 150 ml of milk, micronutrient powder and nutritional counseling for 90 days. A total of 222 participants (LAZ <-1) were included in this analysis on the basis of "response to nutritional intervention". Here, "response to nutritional intervention (improvement)" has been defined by "any positive change of LAZ" at the end line from baseline after receiving nutritional intervention. However, "no change of LAZ" at end line from baseline has been considered as "failure to respond to nutritional intervention (or non-improvement)". Among the participants, 118 failed to respond to nutritional intervention and their linear growth, defined by LAZ score, did not improve (no change of LAZ) at end line from baseline, were considered as "non-improved group". Whereas, 104 participants who responded to nutritional intervention and nutritionally improved (any positive change of LAZ at the end line) were included in "improved group". Study participants were enrolled from Bauniabadh, a slum of Mirpur, Dhaka, Bangladesh. The study protocol (PR-16007) was approved by the institutional review board of International Centre for Diarrheal Disease Research, Bangladesh (icddr,b). Written consent was obtained from mother of the participant after explaining about the study.

Data acquisition, sample collection and laboratory assay

Anthropometric and socio-economic data were collected from study participants by trained staff. Length-for age z-score (LAZ), Weight-for length z-score (WLZ), Weight-for age z-score (WAZ), Mid-upper arm circumference (MUAC) and Head circumference of the study participants were calculated as anthropometric indices. A total of 4 ml of blood was collected by medical doctor following all the aseptic conditions and transported to laboratory maintaining cold condition (-4° c). Plasma was collected by centrifugation of blood at 3000 xg for 10 minutes. Aliquots of biological samples were stored at -80°C until biomarker analyses. Plasma

Confidential: For Review Only

biomarkers including LRP1 (BIOMATIK, USA), sCD14 (R& D system, USA), C-reactive protein (Immundiagnostik, Germany), Alpha-1 acid glycoprotein (Alpco, NH), and ferritin (ORGENTEC, Germany) were measured by using available ELISA kits. Atomic absorption spectrometry was used to measure the level of Zinc in plasma. Stool sample were utilized to detect the level of biomarkers including LCN2 (R&D system, USA), Neopterin (GenWay Biotech, California), Myeloperoxidase (Alpco, New Hampshire), Alpha-1 antitrypsin (Biovendor, North Carolina), Reg1B (TechLab, USA), Calprotectin (BU¨HLMANN, Switzerland) by using available ELISA kit. All the laboratory analyses were performed at the parasitology laboratory of icddr,b.

Statistical analyses

Data analyses were performed to present the characteristics of the study participants. Data were presented as median with inter-quartile ranges when variables show skewed distributions, while categorical variables were showed as frequencies with percentages. The variables showing normal distribution were presented as mean with standard deviation (SD). Mann-Whitney U test was used to detect the differences in the age, family income, plasma biomarkers including C-reactive protein (CRP), Alpha-1-acid glycoprotein (AGP), sCD14, LRP1, ferritin and zinc as well as fecal biomarkers including Myeloperoxidase (MPO), Neopterin (NEO), Alpha-1 antitrypsin (A1AT), Calprotectin, Reg1B and LCN2; whereas Chi-square test was performed for comparing categorical variables of the participants. Student's t-test was performed to see the differences in length, weight, MUAC, head circumference, LAZ, WLZ, WAZ, maternal height and WAMI index (Water, sanitation, hygiene, Asset, Maternal education and Income index, ranging from 0 to 1) [25]. Spearman's correlation coefficient (*r*) was calculated to see the correlation of LRP1 and LCN2 with others fecal biomarkers and EED score (ranging from 0 to 10) that has been calculated from MPO, NEO and A1AT using the method published earlier [26]. A multivariable linear regression was done to investigate the association of LCN2 and LRP1 with biomarkers of inflammation and EED. Moreover, a multivariable logistic regression was performed to explore the associated factors of "failure to respond to nutritional intervention (non-improvement)". Here, failure or respond to nutritional intervention were considered as outcome variable where "failure to respond to nutritional intervention" has been dichotomized as "1" and "respond to nutritional intervention" has been dichotomized as "0". In this analysis variable showing p-value < 0.2 in bivariate analysis were included in multivariable logistic model. In this analysis, p-value <0.05 was considered statistically significant. All the analyses were performed using STATA version -13.

Confidential: For Review Only

Patient and public involvement

No patient or public were involved with the research design, protocol development and participant's enrollment. None of them will be involved during dissemination of findings.

RESULTS

Anthropometric, socioeconomic and biochemical parameters of the study participants

Table-1 presents the anthropometric and socioeconomic characteristics of the participants. Among the participants nearly half were female. The value of weight, length, MUAC, head circumference, WAZ, LAZ, WLZ and family income were significantly higher in nutritionally improved participants.

Table 1 Anthropometric and socioeconomic characteristics of the study participants*

	Overall (n=222)	Non-Improved (n=118)	Improved (n=104)	p-value
Child age (month), Median (Q1,Q2))	18.3 (16.5, 20.3)	18.4 (16.8, 20.2)	18.1 (16.2, 20.3)	0.795
Female sex□, n(%)	116 (52.3)	69 (58.5)	47 (45.2)	0.048
Weight in kg, Mean (SD)	9.05 (0.959)	8.70 (0.908)	9.45 (0.858)	<0.001
Length in cm, Mean (SD)	76.7 (2.85)	75.4 (2.79)	78.0 (2.24)	<0.001
MUAC in cm, Mean (SD)	14.2 (0.934)	13.9 (0.924)	14.5 (0.839)	<0.001
Head circumference in cm, Mean (SD)	44.7 (1.38)	44.4 (1.45)	44.9 (1.25)	0.008
Weight for age z score, Mean (SD)	-1.44 (0.860)	-1.75 (0.865)	-1.09 (0.714)	<0.001
Length for age z score, Mean (SD)	-1.82 (0.799)	-2.21 (0.802)	-1.37 (0.504)	<0.001
Weight for length z score, Mean (SD)	-0.774 (0.916)	-0.923 (0.915)	-0.605 (0.891)	0.009
Maternal height in cm, Mean (SD)	149 (5.09)	149 (5.29)	150 (4.80)	0.068
Family income per month (USD), Median (Q1,Q2)	179 (124, 239)	155 (121, 238)	184 (127, 248)	0.025
Improved sanitation□, n(%)	152 (68.5)	85 (72.0)	67 (64.4)	0.223
WAMI†, Mean (SD)	0.598 (0.134)	0.586 (0.136)	0.612 (0.130)	0.155

** Anthropometric and socioeconomic data, those were collected after nutritional intervention have been used in these analyses*

† WAMI: Water, sanitation, hygiene, Asset, Maternal education and Income (ranging from 0 to 1)

□ Categorical variables (Sex and Improved sanitation)

Confidential: For Review Only

The levels of plasma biomarkers including LRP1, AGP, ferritin and fecal biomarkers including NEO, AAT, Reg1B, and LCN2 were higher in nutritionally improved participants (Table-2). Whereas, plasma level of CRP, sCD14, Zinc as well as fecal biomarkers such as MPO and Calprotectin were lower in nutritionally improved participants.

Table 2 Levels of plasma and fecal biomarkers of the study participants†

	Overall (n=222)	Non-Improved (n=118)	Improved (n=104)	p-value*
Plasma biomarkers				
LRP1 (ng/ml), Median (Q1, Q3)	1080 (796, 1390)	1040 (811, 1370)	1100 (780, 1410)	0.828
AGP (mg/dl), Median (Q1, Q3)	91.9 (69.0, 118)	89.9 (66.9, 119)	95.5 (74.70, 117)	0.449
CRP (mg/L), Median (Q1, Q3)	0.820 (0.284, 2.84)	0.857 (0.274, 2.88)	0.796 (0.30, 2.42)	0.666
sCD14 (ng/ml), Median (Q1, Q3)	1760 (1470, 2120)	1830 (1510, 2220)	1700 (1400, 2000)	0.011
Ferritin (ng/ml), Median (Q1, Q3)	13.8 (7.17, 25.1)	12.7 (6.62, 20.9)	14.7 (7.91, 29.20)	0.065
Zinc (mg/L), Median (Q1, Q3)	0.760 (0.678, 0.840)	0.760 (0.67, 0.85)	0.755 (0.68, 0.83)	0.615
Fecal biomarkers				
Myeloperoxidase (ng/ml), Median (Q1, Q3)	1720 (796, 3720)	1850 (751, 4370)	1570 (881, 3030)	0.435
Neopterin (nmol/L), Median (Q1, Q3)	1240 (567, 2690)	1120 (490, 2490)	1440 (728, 2860)	0.088
AAT (mg/g), Median (Q1, Q3)	0.294 (0.13, 0.52)	0.27 (0.12, 0.50)	0.30 (0.15, 0.53)	0.674
Calprotectin (µg/g), Median (Q1, Q3)	372 (208, 677)	374 (195, 600)	371 (226, 839)	0.248
Reg1B (µg/ml), Median (Q1, Q3)	48.8 (13.9, 88.4)	44.7 (8.54, 88.6)	53.5 (19.4, 87.7)	0.203
LCN 2 (µg/g), Median (Q1, Q3)	134 (41.6, 280)	124 (29.1, 221)	140 (67.1, 317)	0.049

*†Biochemical data, those were measured after nutritional intervention have been used in these analyses; * Mann-Whitney U test*

Correlation and association of LCN 2 and LRP1 with biomarkers and EED score

A significant positive correlation was found among LCN2 and different biomarkers of intestinal inflammation like MPO, NEO, Calprotectin and Reg1B. LCN2 also positively correlated with EED score. However, LRP1 was negatively and significantly correlated with MPO, NEO, A1AT, Reg1B and EED score. Moreover, MPO was found to be positively correlated with NEO, A1AT and Calprotectin. A1AT also positively correlated with Calprotectin. Correlations among different variables are illustrated in table-3.

Confidential: For Review Only

Table 3 Correlation of LCN 2 and LRP1 with others fecal biomarkers and EED score

Variables	LRP1	LCN 2	MPO	NEO	A1AT	Calprotectin	Reg1B	EED score
LRP1	1.00							
LCN 2	-0.04	1.00						
MPO	-0.18*	0.19*	1.00					
NEO	-0.30*	0.20*	0.16*	1.00				
A1AT	-0.18*	-0.005	0.33*	-0.006	1.00			
Calprotectin	-0.12	0.30*	0.55*	0.12	0.25*	1.00		
Reg1B	-0.20*	0.20*	0.10	0.13	-0.03	0.07	1.00	
EED score	-0.29*	0.20*	0.78*	0.37*	0.70*	0.50*	0.08	1.00

**Statistically significant (Exact p-value are shown in supplementary file)*

In this study, multivariable linear regression (Supplementary file, Table-S1) showed a significant positive association of fecal LCN2 with fecal MPO. Whereas, plasma LRP1 was found to be negatively and significantly associated with NEO and Reg1B (Supplementary file, Table-S2 & S3). LRP1 was also found to be negatively associated with MPO and Calprotectin, but not significant.

Factors associated with non- improvement of study participants

Lower level of LCN2 and ferritin as well as lower family income were positively associated with the "failure to respond to nutritional intervention (non-improvement)". Thus, these factors act as protective role against non-improvement. However, female participants were more prone to "failure to respond to nutritional intervention" than male participants. Table-4 presents the results of multivariable logistic regression to investigate the associated factors of non-improvement.

Table 4 Multivariable logistic regression analysis to explore the factors associated with "non-improvement"

	Unadjusted OR (95% CI)	p-value	Adjusted OR (95% CI)	p-value
Child age (month)	1.0005 (0.996, 1.004)	0.794	1.029 (0.894, 1.185)	0.684
Female sex	1.07 (1.003, 2.907)	0.049	1.923 (1.058, 3.493)	0.032
Maternal height (cm)	0.952 (0.903, 1.003)	0.069	0.959 (0.906, 1.016)	0.163
Family income (USD)	0.996 (.994, 0.999)	0.020	0.996 (0.939, 0.999)	0.017
Ferritin (ng/ml)	0.974 (0.955, 0.992)	0.006	0.966 (0.945, 0.987)	0.002
sCD14 (ng/ml)	1.0008 (1.00, 1.001)	0.006	1.001 (1.000, 1.001)	0.004
Neopterin (nmol/L)	0.999 (0.999, 1.000)	0.131	0.999 (0.999, 1.00)	0.140
LCN 2 (µg/g)	0.998 (0.997, 1.000)	0.061	0.998 (0.996, 0.999)	0.036

*Outcome variable: failure (1) or respond (0) to nutritional intervention

https://mc.manuscriptcentral.com/bmjpo

Confidential: For Review Only

DISCUSSION

Our results reveal that LCN2 positively correlates with biomarkers of intestinal inflammation including MPO, NEO, Calprotectin and Reg1B. These results are in accordance with the findings of another study, where LCN2 has been found to be positively correlated with MPO and Calprotectin [27]. Another study also reported positive correlation between LCN2 and Reg1-B, this is also in line with our findings [28]. Moreover, MPO, NEO, Caprotectin have been emerged as a biomarkers of intestinal inflammation as well as predictor of EED [26 29-32]. Since LCN2 correlates with these biomarkers, so it might be a potential biomarker to diagnose intestinal inflammation and EED. Study also reported that LCN2 is released from various cell types and up-regulated in tissue damaging conditions such as ulcerative colitis ,infection and burn injury [6 27]. It has been conceded as an promising biomarker of inflammation, infection, ischemia as well as kidney damage [6]. LCN2 is highly expressed in intestinal epithelial cell in case of inflammatory bowel disease (IBD) [6]. Moreover, it has been reported as a dynamic and sensitive biomarker of gut inflammation [33]. In our analysis LCN2 was also found to be positively associated with MPO. Besides, LCN2 also positively correlated with EED score. Overall, our finding implies that LCN2 might be a potential biomarker for intestinal inflammation as well as it may be used as a predictor of EED.

Our results also showed that LCN2 level and family income are significantly higher in improved group compare to non-improved group. In this study both the groups received nutritional intervention, but a deviation came to light, which reflects that increased linear growth cannot be achieved by nutritional intervention among some participants (who failed to respond to nutritional intervention), while some participants improved after intervention. There might have some factors and rudimentary causes that can play role for this aberration. In this regard, we performed multivariable logistic regression that showed that LCN2, Ferritin and family income acts as a protective role against "failure of response to nutritional intervention (Non-improvement)". Thus, it can be said that higher level of LCN2 and Ferritin as well as higher family income may contribute to get improved upon nutritional intervention. However, lower level of LCN2 and Ferritin as well as lower income may cause failure to respond to nutritional intervention. In addition, LCN2 was reported as a growth factor, differentiation factor and an adipokine in various studies [34-37]. Moreover, it has been suggested to have a role as an appetite regulating hormone [38]. Altogether, these findings proposed that LCN2 may exert protective role against non-improvement by different physiological functions. Larger experimental trail is warranted to find the mechanism by which LCN2 shows its protective role. Study showed that children of lower income household

Confidential: For Review Only

are more likely to be stunted and conclude that families with higher income have more capability to provide nutritional requirements for their children than lower income families [39], thus higher family income play protective role against non-improvement. Whereas, female gender was found to be more susceptible for being failed to get nutritionally improved in our study. Study reported that female are more likely to be undernourished compare to male in Bangladesh [40]. This is the first study to examine the association of these factors with nutritional improvement.

In our analysis we also measured the level of plasma LRP1. Correlation among the plasma LRP1 and fecal biomarkers showed that LRP1 is negatively correlated with MPO, NEO, AAT and Reg1B. Moreover, LRP1 was found to be negatively associated with NEO and Reg1B. Moreover, LRP1 also negatively correlates with EED score. MPO, NEO, A1AT have been reported as biomarkers of intestinal inflammation and EED [26 29-31] and correlates negatively with LRP1. Therefore, these results support our supposition as we hypothesized that LRP1 may reduce intestinal inflammation. Overall, it can be inferred that LRP1 may contribute in the reduction of intestinal inflammation. Our finding is supported by another study, where it has been reported that LRP1 exerts anti-inflammatory effect upon LPS exposure [41]. LRP1 also functions in activation of lysosomal enzymes, phagocytosis as well as destruction of microorganisms thus LRP1 contributes in reduction of gut inflammation [16 19 42]. LRP1 was also found to be played a pivotal role in elimination of pathogen by scavenging membrane lipoproteins of microorganism during microbial invasion in another study [23]. On the other hand, intestinal inflammation has been observed in LRP1 knockout mice [4]. Altogether, these findings strongly suggest that LRP1 has an effect on the reduction of gut inflammation. Our study results also reported that, MPO is positively and significantly correlated with NEO, A1AT and Calprotectin. These findings are in accordance with other studies [26 27], where MPO was found to be positively correlated with these biomarkers. A1AT was also found to be positively correlated with calprotectin in our study, which is in alignment with other finding [43].

Limitations and strengths

There were several limitations in our study. Firstly, we could not measure the genetic expression of LCN2 and LRP1 of participants due to invasive collection process of intestinal cell. Secondly, we did not examine the consequence of suppression of LRP1 expression of human to investigate whether decreased expression may or may not contribute to gut inflammation because of ethical consideration. Lack of age and sex matched healthy children was also a limitation in this study. However, this was the first study that investigated the relationship of

Confidential: For Review Only

LCN2 and LRP1 with biomarkers of EED and examined the associated factors of non-improvement of participants which are the strengths of this study.

CONCLUSIONS

Our findings suggest that LCN2 might be a potential biomarker to predict the gut inflammation and EED. However, increased level of LRP1 may contribute in alleviating intestinal inflammation that could be a potential therapeutic target to reduce the inflammation.

Acknowledgments We cordially thank all the participants and their mothers of the BEED study as well as the laboratory and field staffs at icddr,b for their contributions. icddr,b also gratefully acknowledges the donors (Bill & Melinda Gates Foundation) of this research protocol.

Contributors TA conceptualized the study. MMH wrote the manuscript and analyzed the data. MMH, MAG, FH did the laboratory analysis. MAG, SD, SMF critically reviewed the manuscript. MAA, MMH involved in statistical analysis. MM, TA involved in the development of study protocol. All authors commented on manuscript and approved the final version.

Funding This study was funded by the Bill & Melinda Gates Foundation. The investment ID was OPP1136751.(https://www.gatesfoundation.org/How-We-Work/Quick-Links/GrantsDatabase/Grants/2015/11/OPP1136751).

Competing interests The authors have no competing interest.

Patient consent for publication Written consent was collected from mother of the participants during enrollment.

Ethics approval The study protocol was approved by Institutional review board of the International Centre for Diarrhoeal Disease Research, Bangladesh (icddr,b). Protocol number-PR-16007.

Provenance and peer review Not commissioned; peer reviewed externally.

Data availability statement The data set that was created during the study is not publicly available due to the restriction of funder. However, suggestion for data analysis can be made to corresponding author.

https://mc.manuscriptcentral.com/bmjpo

Confidential: For Review Only

REFERENCES

1. Korpe PS, Petri Jr WA. Environmental enteropathy: critical implications of a poorly understood condition. *Trends in molecular medicine* 2012;18(6):328-36.
2. Lunn P, Northrop-Clewes C, Downes R. Intestinal permeability, mucosal injury, and growth faltering in Gambian infants. *The Lancet* 1991;338(8772):907-10.
3. Fahim SM, Das S, Sanin KI, et al. Association of Fecal Markers of Environmental Enteric Dysfunction with Zinc and Iron Status among Children at First Two Years of Life in Bangladesh. *The American journal of tropical medicine and hygiene* 2018;99(2):489-94.
4. Uchiyama R, Kupkova K, Shetty SJ, et al. Histone H3 lysine 4 methylation signature associated with human undernutrition. *Proceedings of the National Academy of Sciences* 2018;115(48):E11264-E73.
5. Flo TH, Smith KD, Sato S, et al. Lipocalin 2 mediates an innate immune response to bacterial infection by sequestrating iron. *Nature* 2004;432(7019):917-21.
6. Moschen AR, Adolph TE, Gerner RR, et al. Lipocalin-2: a master mediator of intestinal and metabolic inflammation. *Trends in Endocrinology & Metabolism* 2017;28(5):388-97.
7. Toyonaga T, Matsuura M, Mori K, et al. Lipocalin 2 prevents intestinal inflammation by enhancing phagocytic bacterial clearance in macrophages. *Scientific reports* 2016;6:35014.
8. Flower DR, North AC, Sansom CE. The lipocalin protein family: structural and sequence overview. *Biochimica et Biophysica Acta (BBA)-Protein Structure and Molecular Enzymology* 2000;1482(1-2):9-24.
9. Schaible UE, Kaufmann SH. Iron and microbial infection. *Nature Reviews Microbiology* 2004;2(12):946-53.
10. Goetz DH, Holmes MA, Borregaard N, et al. The neutrophil lipocalin NGAL is a bacteriostatic agent that interferes with siderophore-mediated iron acquisition. *Molecular cell* 2002;10(5):1033-43.
11. Chan YR, Liu JS, Pociask DA, et al. Lipocalin 2 is required for pulmonary host defense against Klebsiella infection. *The Journal of Immunology* 2009;182(8):4947-56.
12. Nairz M, Schroll A, Haschka D, et al. Lipocalin-2 ensures host defense against Salmonella Typhimurium by controlling macrophage iron homeostasis and immune response. *European journal of immunology* 2015;45(11):3073-86.
13. Wu H, Santoni-Rugiu E, Ralfkiaer E, et al. Lipocalin 2 is protective against E. coli pneumonia. *Respiratory research* 2010;11(1):96.
14. Yammine L, Zablocki A, Baron W, et al. Lipocalin-2 Regulates Epidermal Growth Factor Receptor Intracellular Trafficking. *Cell reports* 2019;29(7):2067-77. e6.
15. Ronco C, Legrand M, Goldstein SL, et al. Neutrophil gelatinase-associated lipocalin: ready for routine clinical use? An international perspective. *Blood purification* 2014;37(4):271-85.
16. Lillis AP, Van Duyn LB, Murphy-Ullrich JE, et al. LDL receptor-related protein 1: unique tissue-specific functions revealed by selective gene knockout studies. *Physiological reviews* 2008;88(3):887-918.
17. Gonias SL, Campana WM. LDL receptor–related protein-1: a regulator of inflammation in atherosclerosis, cancer, and injury to the nervous system. *The American journal of pathology* 2014;184(1):18-27.
18. Strickland DK, Gonias SL, Argraves WS. Diverse roles for the LDL receptor family. *Trends in Endocrinology & Metabolism* 2002;13(2):66-74.
19. Lim JJ, Grinstein S, Roth Z. Diversity and versatility of phagocytosis: roles in innate immunity, tissue remodeling, and homeostasis. *Frontiers in cellular and infection microbiology* 2017;7:191.
20. Lin L, Hu K. LRP-1: functions, signaling and implications in kidney and other diseases. *International journal of molecular sciences* 2014;15(12):22887-901.
21. Potere N, Del Buono MG, Mauro AG, et al. Low density lipoprotein receptor-related protein-1 in cardiac inflammation and infarct healing. *Frontiers in cardiovascular medicine* 2019;6:51.
22. Yancey PG, Blakemore J, Ding L, et al. Macrophage LRP-1 controls plaque cellularity by regulating efferocytosis and Akt activation. *Arteriosclerosis, thrombosis, and vascular biology* 2010;30(4):787-95.
23. May P. The low-density lipoprotein receptor-related protein 1 in inflammation. *Current opinion in lipidology* 2013;24(2):134-37.
24. Mahfuz M, Das S, Mazumder RN, et al. Bangladesh environmental enteric dysfunction (BEED) study: protocol for a community-based intervention study to validate non-invasive biomarkers of environmental enteric dysfunction. *BMJ open* 2017;7(8):e017768.
25. Pradhan NA, Ali TS, Hasnani FB, et al. Measuring socio-economic status of an urban squatter settlement in Pakistan using WAMI Index. *J Pak Med Assoc* 2018;68:709-14.

Confidential: For Review Only

26. Kosek M, Haque R, Lima A, et al. Fecal markers of intestinal inflammation and permeability associated with the subsequent acquisition of linear growth deficits in infants. *The American journal of tropical medicine and hygiene* 2013;88(2):390-96.
27. Prata MdMG, Havt A, Bolick D, et al. Comparisons between myeloperoxidase, lactoferrin, calprotectin and lipocalin-2, as fecal biomarkers of intestinal inflammation in malnourished children. *Journal of translational science* 2016;2(2):134.
28. Guerrant RL, Leite AM, Pinkerton R, et al. Biomarkers of environmental enteropathy, inflammation, stunting, and impaired growth in children in northeast Brazil. *PloS one* 2016;11(9):e0158772.
29. George CM, Oldja L, Biswas SK, et al. Fecal markers of environmental enteropathy are associated with animal exposure and caregiver hygiene in Bangladesh. *The American journal of tropical medicine and hygiene* 2015;93(2):269-75.
30. Arndt MB, Richardson BA, Ahmed T, et al. Fecal markers of environmental enteropathy and subsequent growth in Bangladeshi children. *The American journal of tropical medicine and hygiene* 2016;95(3):694-701.
31. George CM, Oldja L, Biswas S, et al. Geophagy is associated with environmental enteropathy and stunting in children in rural Bangladesh. *The American journal of tropical medicine and hygiene* 2015;92(6):1117-24.
32. Konikoff MR, Denson LA. Role of fecal calprotectin as a biomarker of intestinal inflammation in inflammatory bowel disease. *Inflammatory bowel diseases* 2006;12(6):524-34.
33. Chassaing B, Srinivasan G, Delgado MA, et al. Fecal lipocalin 2, a sensitive and broadly dynamic non-invasive biomarker for intestinal inflammation. *PloS one* 2012;7(9):e44328.
34. Chakraborty S, Kaur S, Guha S, et al. The multifaceted roles of neutrophil gelatinase associated lipocalin (NGAL) in inflammation and cancer. *Biochimica et Biophysica Acta (BBA)-Reviews on Cancer* 2012;1826(1):129-69.
35. Devireddy LR, Gazin C, Zhu X, et al. A cell-surface receptor for lipocalin 24p3 selectively mediates apoptosis and iron uptake. *Cell* 2005;123(7):1293-305.
36. Wang Y, Lam KS, Kraegen EW, et al. Lipocalin-2 is an inflammatory marker closely associated with obesity, insulin resistance, and hyperglycemia in humans. *Clinical chemistry* 2007;53(1):34-41.
37. Yan Q-W, Yang Q, Mody N, et al. The adipokine lipocalin 2 is regulated by obesity and promotes insulin resistance. *Diabetes* 2007;56(10):2533-40.
38. Mosialou I, Shikhel S, Liu J-M, et al. MC4R-dependent suppression of appetite by bone-derived lipocalin 2. *Nature* 2017;543(7645):385-90.
39. Sultana P, Rahman MM, Akter J. Correlates of stunting among under-five children in Bangladesh: a multilevel approach. *BMC nutrition* 2019;5(1):1-12.
40. Pryer JA, Rogers S. Epidemiology of undernutrition in adults in Dhaka slum households, Bangladesh. *European journal of clinical nutrition* 2006;60(7):815-22.
41. Luo L, Wall AA, Tong SJ, et al. TLR crosstalk activates LRP1 to recruit Rab8a and PI3Kγ for suppression of inflammatory responses. *Cell reports* 2018;24(11):3033-44.
42. Safina D, Schlitt F, Romeo R, et al. Low-density lipoprotein receptor-related protein 1 is a novel modulator of radial glia stem cell proliferation, survival, and differentiation. *Glia* 2016;64(8):1363-80.
43. Welle I, Bakke P, Eide G, et al. Increased circulating levels of α1-antitrypsin and calprotectin are associated with reduced gas diffusion in the lungs. *European Respiratory Journal* 2001;17(6):1105-11.

Confidential: For Review Only

Table-S1: Multivariable linear regression analysis to see the association between fecal LCN2 and MPO

	Unadjusted β (95% CI)	p-value	Adjusted β (95% CI)	p-value
Lipocalin 2	3.0 (0 .31, 5.81)	0.02	2.9 (0.15, 5.65)	**0.03**
AGP	8.0 (-1.64, 17.84)	0.10	6.08 (-5.01, 17.19)	0.28
CRP	90.76 (-.8876, 182.42)	0.05	71.59 (-33.65, 176.84)	0.18
Ferritin	-25.24 (-58.13, 7.65)	0.13	-32.70 (-66.90, 1.49)	0.06
sCD14	0.81 (-0.28, 1.90)	0.14	0.28 (-.88, 1.45)	0.63
Length	-133.33 (-311.54, 44.94)	0.14	-104.02 (-294.33, 86.29)	0.28
MUAC	-395.56 (-939.53, 148.41)	0.15	-152.56 (-737.42, 432.29)	0.60

Table-S2: Multivariable linear regression analysis to see the association between plasma LRP1 and NEO

	Unadjusted β (95% CI)	p-value	Adjusted β (95% CI)	p-value
LRP1	-0.81 (-1.47, -0.16)	0.01	-0.78 (-1.45, -0.10)	**0.02**
AGP	-3.39 (-8.89, 2.10)	0.22	-5.78 (-12.11, 0.54)	0.07
CRP	20.67 (-31.28, 72.63)	0.43	36.77 (-23.15, 96.71)	0.22
Ferritin	2.98 (-15.62, 21.58)	0.75	7.71 (-11.88, 27.32)	0.43
sCD14	0.22 (-.39, 0.84)	0.48	0.22 (-0.44, 0.89)	0.50
Length	18.04 (-82.83, 118.93)	0.72	28.76 (-80.27, 137.80)	0.60
MUAC	-395.56 (-939.53, 148.41)	0.15	-126.23 (-461.45, 208.97)	0.45

Table-S3: Multivariable linear regression analysis to examine the association between LRP1 and Reg1B

	Unadjusted β (95% CI)	p-value	Adjusted β (95% CI)	p-value
LRP1	-0.03 (-0.06, -0.01)	0.004	-0.04 (-0.07, -0.01)	**0.002**
AGP	0.05 (-.17, 0.27)	0.65	0.144 (-0.11, 0.40)	0.26
CRP	-0.94 (-3.06, 1.17)	0.38	-1.18 (-3.62, 1.24)	0.33
Ferritin	0.17 (-.58, 0.93)	0.64	0.31 (-0.47, 1.11)	0.43
sCD14	-0.006 (-.03, 0.01)	0.61	-0.007 (-.03, 0.01)	0.57
Length	1.11 (-2.99, 5.22)	0.59	-0.65 (-5.08, 3.78)	0.77
MUAC	4.58 (-7.95, 17.12)	0.47	6.91 (-6.71, 20.54)	0.31

Confidential: For Review Only

Exact p- value for "**Table 3** Correlation of LCN 2 and LRP1 with others fecal biomarkers and EED score"

A significant positive correlation was found among LCN2 and different biomarkers of intestinal inflammation like MPO (r = 0.19, **p = 0.005**), NEO (r = 0.20, **p = 0.004**), Calprotectin (r= 0.3, **p = 0.0001**), and Reg1B (r = 0.20, **p = 0.003**). LCN2 also positively correlated with EED score (r = 0.20, **p = 0.003**). On the other hand, LRP1 was negatively correlated with MPO (r = - 0.18, **p = 0.006**), NEO (r = - 0.30, **p = 0.0001**), A1AT (r = - 0.18**, p = 0.006**), Reg1B (r= -0.2, **p = 0.003**) as well as EED score (r = - 0.29, **p = 0.0001**). Moreover, MPO was found to be positively correlated with NEO (r= 0.16, **p< 0.05**), A1AT (r= 0.33, **p<0.05**) and Calprotectin (r= 0.55, p<0.05). A1AT also positively correlated with Calprotectin (r=0.25, **p<0.05**).

Confidential: For Review Only

BMJ Paediatrics Open

Association of Lipocalin-2 and Low-density lipoprotein receptor related protein-1 (LRP1) with biomarkers of Environmental Enteric Dysfunction (EED) among under 2 children in Bangladesh: Results from a community based intervention study

Journal:	*BMJ Paediatrics Open*
Manuscript ID	bmjpo-2021-001138.R1
Article Type:	Original research
Date Submitted by the Author:	27-Jun-2021

Complete List of Authors:	Hasan, Md. Mehedi ; International Centre for Diarrhoeal Disease Research Bangladesh<br>Gazi, Md Amran; International Centre for Diarrhoeal Disease Research Bangladesh, NCSD<br>Das, Subhasish; International Centre for Diarrhoeal Disease Research Bangladesh<br>Fahim, Shah Mohammad ; International Centre for Diarrhoeal Disease Research Bangladesh<br>Hossaini, Farzana ; International Centre for Diarrhoeal Disease Research Bangladesh<br>Alam, Md. Ashraful ; International Centre for Diarrhoeal Disease Research Bangladesh,<br>Mahfuz, Mustafa; International Centre for Diarrhoeal Disease Research Bangladesh<br>Ahmed, Tahmeed; International Centre for Diarrhoeal Disease Research, Bangladesh (icddr,b), Clinical Sciences Division (CSD); Centre for Nutrition and Food Security (CNFS)

Keywords:	Biochemistry, Gastroenterology, Growth

SCHOLARONE™
Manuscripts

I, the Submitting Author has the right to grant and does grant on behalf of all authors of the Work (as defined in the below author licence), an exclusive licence and/or a non-exclusive licence for contributions from authors who are: i) UK Crown employees; ii) where BMJ has agreed a CC-BY licence shall apply, and/or iii) in accordance with the terms applicable for US Federal Government officers or employees acting as part of their official duties; on a worldwide, perpetual, irrevocable, royalty-free basis to BMJ Publishing Group Ltd ("BMJ") its licensees and where the relevant Journal is co-owned by BMJ to the co-owners of the Journal, to publish the Work in this journal and any other BMJ products and to exploit all rights, as set out in our licence.

The Submitting Author accepts and understands that any supply made under these terms is made by BMJ to the Submitting Author unless you are acting as an employee on behalf of your employer or a postgraduate student of an affiliated institution which is paying any applicable article publishing charge ("APC") for Open Access articles. Where the Submitting Author wishes to make the Work available on an Open Access basis (and intends to pay the relevant APC), the terms of reuse of such Open Access shall be governed by a Creative Commons licence – details of these licences and which Creative Commons licence will apply to this Work are set out in our licence referred to above.

Other than as permitted in any relevant BMJ Author's Self Archiving Policies, I confirm this Work has not been accepted for publication elsewhere, is not being considered for publication elsewhere and does not duplicate material already published. I confirm all authors consent to publication of this Work and authorise the granting of this licence.

Confidential: For Review Only

Association of Lipocalin-2 and Low-density lipoprotein receptor related protein-1 (LRP1) with biomarkers of Environmental Enteric Dysfunction (EED) among under 2 children in Bangladesh: Results from a community based intervention study

Md. Mehedi Hasan[1], Md. Amran Gazi[1]*, Subhasish Das[1], Shah Mohammad Fahim[1], Farzana Hossaini[1], Md. Ashraful Alam [1], Mustafa Mahfuz[1], Tahmeed Ahmed[1]

[1]Nutrition and Clinical Services Division, International Centre for Diarrheal Disease Research, Bangladesh (icddr,b), Dhaka, Bangladesh

Authors' addresses:

1. Md. Mehedi Hasan, MSc , Senior Research Officer, Nutrition and Clinical Services Division, icddr,b, Dhaka, Bangladesh, Email: md.hasan@icddrb.org
2. Md. Amran Gazi, MSc, Assistant Scientist, Nutrition and Clinical Services Division, icddr,b, Dhaka, Bangladesh, Email: amran.gazi@icddrb.org
3. Subhasish Das, MBBS, MPH, Assistant Scientist, Nutrition and Clinical Services Division, icddr,b, Dhaka, Bangladesh, Email: subhasish.das@icddrb.org
4. Shah Mohammad Fahim, MBBS, MPH; Research Investigator, Nutrition and Clinical Services Division, icddr,b, Dhaka, Bangladesh; Email: mohammad.fahim@icddrb.org
5. Farzana Hossaini, MSc, Senior Research Assistant, Nutrition and Clinical Services Division, icddr,b, Dhaka, Bangladesh, Email: farzanasacky@gmail.com
6. Md. Ashraful Alam, MSc, Assistant Scientist, Nutrition and Clinical Services Division, icddr,b, Dhaka, Bangladesh, Email: mashraful@icddrb.org
7. Mustafa Mahfuz, MBBS, MPH, Associate Scientist, Nutrition and Clinical Services Division, icddr,b, Dhaka, Bangladesh, Email: mustafa@icddrb.org
8. Tahmeed Ahmed, MBBS, PhD, Executive Director, icddr,b, Dhaka, Bangladesh, Email: tahmeed@icddrb.org

***Correspondence to: Md. Amran Gazi,** Nutrition and Clinical Services Division, International Centre for Diarrhoeal Disease Research (icddr,b), Dhaka-1212, Bangladesh, **Email:** amran.gazi@icddrb.org

Word count: Abstract: 234; Main text: 1860

Number of table: 3

Confidential: For Review Only

ABSTRACT

Background Environmental enteric dysfunction (EED) is thought to occur from persistent intestinal inflammation. Studies also revealed the association of Lipocalin-2 (LCN2) and Low-density lipoprotein receptor related protein-1 (LRP1) with intestinal inflammation. Therefore, we intended to explore the relationship of LCN2 and LRP1 with gut inflammation and biomarkers of EED in Bangladeshi malnourished children.

Methods A total of 222 children (Length-for-age z-score (LAZ) <−1) aged 12-18 months were enrolled and provided nutrition intervention for 90 days. Among the participants, 115 were stunted (LAZ <-2) and 107 were at risk of being stunted (LAZ -1 to -2) children. Plasma and fecal biomarkers were measured using ELISA. Spearman's rank correlation was done to see the correlation among LCN2, LRP1 and biological biomarkers.

Results LCN2 correlates positively with Myeloperoxidase (r = 0.19, p = 0.005), Neopterin (r = 0.20, p = 0.004), Calprotectin (r= 0.3, p = 0.0001), Reg1B (r = 0.20, p = 0.003) and EED score (r = 0.20, p = 0.003). Whereas, LRP1 correlates negatively with Myeloperoxidase (r = - 0.18, p = 0.006), Neopterin (r = - 0.30, p = 0.0001), Alpha-1-antitrypsin (r = - 0.18, p = 0.006), Reg1B (r= -0.2, p = 0.003) and EED score (r = - 0.29, p = 0.0001).

Conclusions Our findings imply that LCN2 might be a promising biomarker to predict gut inflammation and EED. Whereas, increased level of LRP1 may contribute to alleviating intestinal inflammation.

Key words: Lipocalin-2, LRP1, gut inflammation, EED, Bangladesh

Confidential: For Review Only

What is known about the subject?

- Environmental enteric dysfunction (EED) is highly prevalent in developing countries which affects approximately 40% of all children with age less than 5 years.
- EED causes linear growth faltering or stunting which has the manifestations including reduced neurodevelopmental and cognitive function and increased morbidity and mortality in childhood.
- The main challenge to tackle the EED is lack of non-invasive biomarker signature to diagnose the EED and take proper treatment initiative.

What this study add?

- There are significant positive correlation of Lipocalin-2 and significant negative correlation of LRP-1 with biomarkers of EED.
- Study findings strongly infer that LCN2 might be a promising biomarker to predict gut inflammation and EED
- Study findings also imply that LRP1 could be a therapeutic target to alleviate intestinal inflammation.

https://mc.manuscriptcentral.com/bmjpo

INTRODUCTION

Environmental enteric dysfunction (EED) is a subacute intestinal inflammation highly prevalent in developing countries which is defined by crypt hyperplasia, villous atrophy, reduction of mucus layer and infiltration of lymphocytes in lamina propria [1]. EED affects approximately 40% of all children with age less than 5 years in resource poor settings and causes linear growth faltering or stunting [2]. It is thought to occur from repeated enteric infections due to poor hygiene and unsanitary environmental conditions, which in turn results in intestinal inflammation, a major driver of occurring EED [3]. Studies have elicited the relation of Lipocalin-2 (LCN2) and low-density lipoprotein receptor-related protein-1(LRP1) with intestinal inflammation. LRP1 knockout has been found to be associated with increased intestinal inflammation in animal model [4], and LCN2-deficient mice are more prone to bacterial infection [5]. On the other hand, higher expression of LCN2 has been reported in various inflammatory conditions [6]. Hence, it is necessary to explore the relation of LCN2 and LRP1 with intestinal inflammation in the context of EED.

LCN2 is a bacteriostatic peptide, a member of lipocalin super family, known as neutrophil gelatinase B-associated lipocalin (NGAL) [7 8]. One of the most recognized functions of LCN2 is to prevent the bacterial uptake of iron crucial for their growth [9 10]. It has been reported that LCN2-deficient mice shows higher mortality rates following infection with *Escherichia coli* than wild type [5]. Study also reported that LCN2 shows preventive mechanism against intestinal inflammation through bacterial clearance by phagocytosis in macrophages [7]. Moreover, it exerts defensive mechanism against *Salmonella typhimurium, Klebsiella pneumoniae* and *E. coli* in different studies [11-13]. Besides the defensive mechanism, LCN2 has emerged as promising biomarker of inflammation. It has been reported as hallmark for the onset of kidney diseases as well as indicator of inflammation in various diseases [6 14 15]. However, there is no study that has conducted to investigate the level of LCN2 in Bangladeshi malnourished children. Therefore, the relation of LCN2 with intestinal inflammation and EED are yet to be elucidated.

LRP1 is an endocytic receptor that facilitates the binding and endocytic transportation of a broad range of biological ligands as well as various molecules function in immune system [16-18]. It has a vital role in phagocytosis which is an essential component of the innate immune response, crucial for removal of infectious agents [16 19]. Moreover, LRP1 promotes tissue-resident macrophage-survival by activating Akt pathway [20]. Macrophages show the initial defensive mechanism by eliminate noxious agents including bacteria, irritants and necrotic cells [21], whereas LRP1 deficiency causes increased macrophage apoptosis [22]. LRP1 also mediates

https://mc.manuscriptcentral.com/bmjpo

Confidential: For Review Only

pathogen elimination by scavenging membrane lipoproteins of microorganism during infection [23]. Therefore, it can be hypothesized that LRP1 may reduce the gut inflammation by showing defensive mechanism against microbial invasion. In this study, we intended to investigate the level of LCN2 and LRP1 in malnourished children of Bangladesh and their relations with fecal markers of intestinal inflammation and EED.

METHODS

Study participants, nutritional intervention, study site and ethical consideration

To perform the analysis for this study, data were taken from "Bangladesh Environmental Enteric Dysfunction (BEED)" study. The study protocol was published earlier [24]. It is a community based intervention study to validate noninvasive biomarkers of EED. The intervention package included an egg, 150 ml of milk, micronutrient powder and nutritional counseling. According to the objective of the BEED study, firstly participants were enrolled and their "baseline" data were collected during enrolment and these participants were given nutritional intervention for 90 days, this intervention were started immediately after baseline data collection. After completion of the intervention the "end line" data were collected from the participants. A total of 222 participants were included in this analysis, among them 115 were stunted (LAZ <-2) and 107 were at risk of being stunted (LAZ -1 to -2). Study participants were enrolled from Bauniabadh, a slum of Mirpur, Dhaka, Bangladesh. The study protocol (PR-16007) was approved by the institutional review board of International Centre for Diarrheal Disease Research, Bangladesh (icddr,b). Written consent was obtained from mother of the participant after explaining about the study.

Data acquisition, sample collection and laboratory assay

Anthropometric and socio-economic data were collected from study participants by trained staff. Length-for age z-score (LAZ), Weight-for length z-score (WLZ), Weight-for age z-score (WAZ), Mid-upper arm circumference (MUAC) and Head circumference of the study participants were calculated as anthropometric indices. A total of 4 ml of blood was collected by medical doctor following all the aseptic conditions and transported to laboratory maintaining cold condition (-4° c). Plasma was collected by centrifugation of blood at 3000 xg for 10 minutes. Aliquots of biological samples were stored at -80°C until biomarker analyses. Plasma biomarkers including LRP1 (BIOMATIK, USA), sCD14 (R& D system, USA), C-reactive protein (Immundiagnostik, Germany), Alpha-1 acid glycoprotein (Alpco, NH), and ferritin (ORGENTEC, Germany) were measured by using available ELISA kits. Atomic absorption spectrometry was used to measure the level

https://mc.manuscriptcentral.com/bmjpo

Confidential: For Review Only

of Zinc in plasma. Stool sample were utilized to detect the level of biomarkers including LCN2 (R&D system, USA), Neopterin (GenWay Biotech, California), Myeloperoxidase (Alpco, New Hampshire), Alpha-1 antitrypsin (Biovendor, North Carolina), Reg1B (TechLab, USA), Calprotectin (BU¨HLMANN, Switzerland) by using available ELISA kit. All the laboratory analyses were performed at the parasitology laboratory of icddr,b.

Statistical analyses

Data analyses were performed to present the characteristics of the study participants. Data were presented as median with inter-quartile ranges when variables show skewed distributions, while categorical variables were showed as frequencies with percentages. The variables showing normal distribution were presented as mean with standard deviation (SD). Mann-Whitney U test was used to detect the differences in the age, family income, plasma biomarkers including C-reactive protein (CRP), Alpha-1-acid glycoprotein (AGP), sCD14, LRP1, ferritin and zinc as well as fecal biomarkers including Myeloperoxidase (MPO), Neopterin (NEO), Alpha-1 antitrypsin (A1AT), Calprotectin, Reg1B and LCN2; whereas Chi-square test was performed for comparing categorical variables of the participants. Student's t-test was performed to see the differences in length, weight, MUAC, head circumference, LAZ, WLZ, WAZ, maternal height and WAMI index (Water, sanitation, hygiene, Asset, Maternal education and Income index, ranging from 0 to 1) [25]. Spearman's correlation coefficient (*r*) was calculated to see the correlation of LRP1 and LCN2 with others fecal biomarkers and EED score (ranging from 0 to 10) that has been calculated from MPO, NEO and A1AT using the method published earlier [26]. In this analysis correlation was done taking the end line data (data those were collected after nutritional intervention) of the fecal biomarkers. In this analysis, p-value <0.05 was considered statistically significant. All the analyses were performed using STATA version -13.

Patient and public involvement

No patient or public were involved with the research design, protocol development and participant's enrollment. None of them will be involved during dissemination of findings.

RESULTS

Anthropometric, socioeconomic and biochemical parameters of the study participants

Table-1 presents the anthropometric and socioeconomic characteristics of the participants. Among the participants nearly half were female. The value of weight, length, MUAC, head circumference, WAZ, LAZ and

https://mc.manuscriptcentral.com/bmjpo

Confidential: For Review Only

WLZ were comparatively higher in end line than baseline. Data about sex, maternal height, family income, improved sanitation and WAMI were collected at only one-time point (Baseline).

Table 1 Anthropometric and socioeconomic characteristics of the study participants

	Baseline (n=222)	End line (n=222)
Child age (month), Median (Q1,Q2))	15.4 (13.2, 17.4)	18.3 (16.5, 20.3)
Female sex□, n(%)	116 (52.3)	-
Weight in kg, Mean (SD)	8.32 (0.90)	9.05 (0.96)
Length in cm, Mean (SD)	72.69 (2.75)	76.7 (2.85)
MUAC in cm, Mean (SD)	13.84 (0.90)	14.2 (0.93)
Head circumference in cm, Mean (SD)	44 (1.4)	44.7 (1.38)
Weight for age z score, Mean (SD)	-1.55 (0.88)	-1.44 (0.86)
Length for age z score, Mean (SD)	-2.03 (0.76)	-1.82 (0.79)
Weight for length z score, Mean (SD)	-0.78 (0.93)	-0.774 (0.91)
Maternal height in cm, Mean (SD)	149 (5.09)	-
Family income per month (USD),Median (Q1,Q2)	179 (124, 239)	-
Improved sanitation□, n(%)	152 (68.5)	-
WAMI†, Mean (SD)	0.598 (0.134)	-

† WAMI: Water, sanitation, hygiene, Asset, Maternal education and Income (ranging from 0 to 1)

□ Categorical variables (Sex and Improved sanitation)

Baseline data: data those were collected before nutritional intervention; End line data: data those were collected after nutritional intervention

The levels of plasma biomarkers including LRP1, AGP, CRP, Zinc and fecal biomarkers including MPO, NEO, AAT, Calprotectin and Reg1B, were comparatively lower in end line than baseline (Table-2). LCN2 were measured at only one-time point (End line).

Confidential: For Review Only

Table 2 Levels of plasma and fecal biomarkers of the study participants

	Baseline (n=222)	End line (n=222)
Plasma biomarkers		
LRP1 (ng/ml), Median (Q1, Q3)	1180 (825, 1532)	1080 (796, 1390)
AGP (mg/dl), Median (Q1, Q3)	107.5 (81.3, 37.1)	91.9 (69.0, 118)
CRP (mg/L), Median (Q1, Q3)	1.49 (0.38, 5.72)	0.820 (0.284, 2.84)
sCD14 (ng/ml), Median (Q1, Q3)	1691.38 (1398.2, 2036.02)	1760 (1470, 2120)
Ferritin (ng/ml), Median (Q1, Q3)	13.26 (6.3, 25.84)	13.8 (7.17, 25.1)
Zinc (mg/L), Median (Q1, Q3)	0.78 (0.69, 0.87)	0.760 (0.68, 0.84)
Fecal biomarkers		
MPO (ng/ml), Median (Q1, Q3)	2262 (1191.5, 5646)	1720 (796, 3720)
Neopterin (nmol/L), Median (Q1, Q3)	1844 (811, 3122)	1240 (567, 2690)
AAT (mg/g), Median (Q1, Q3)	0.36 (0.14, 0.67)	0.294 (0.13, 0.52)
Calprotectin (µg/g), Median (Q1, Q3)	491.73 (275.14, 1025.11)	372 (208, 677)
Reg1B (µg/ml), Median (Q1, Q3)	67.28 (35, 93.38)	48.8 (13.9, 88.4)
LCN 2 (µg/g), Median (Q1, Q3)	-	134 (41.6, 280)

Correlation of LCN 2 and LRP1 with biomarkers and EED score

A significant positive correlation was found among LCN2 and different biomarkers of intestinal inflammation like MPO, NEO, Calprotectin and Reg1B. LCN2 also positively correlated with EED score. However, LRP1 was negatively and significantly correlated with MPO, NEO, A1AT, Reg1B and EED score. Correlations among different variables are illustrated in table-3.

Table 3 **Correlation of LCN 2 and LRP1 with others fecal biomarkers and EED score

Variables	LRP1	LCN 2
LRP1	1.00	
LCN 2	-0.04	1.00
MPO	-0.18*	0.19*
NEO	-0.30*	0.20*
A1AT	-0.18*	-0.005
Calprotectin	-0.12	0.30*
Reg1B	-0.20*	0.20*
EED score	-0.29*	0.20*

**Statistically significant (Exact p-value are shown in supplementary file)*

***Correlation was done taking the end line data (data those were collected after nutritional intervention) of the fecal biomarkers*

Confidential: For Review Only

DISCUSSION

Our results reveal that LCN2 positively correlates with biomarkers of intestinal inflammation including MPO, NEO, Calprotectin and Reg1B. These results are in accordance with the findings of another study, where LCN2 has been found to be positively correlated with MPO and Calprotectin [27]. Another study also reported positive correlation between LCN2 and Reg1-B, this is also in line with our findings [28]. Moreover, MPO, NEO, Caprotectin have been emerged as a biomarkers of intestinal inflammation as well as predictor of EED [26 29-32]. Therefore, LCN2 might be a potential biomarker to diagnose intestinal inflammation and EED. Study also reported that LCN2 is released from various cell types and up-regulated in tissue damaging conditions such as ulcerative colitis, infection and burn injury [6 27]. It has been conceded as an promising biomarker of inflammation, infection, ischemia as well as kidney damage [6]. LCN2 is highly expressed in intestinal epithelial cell in case of inflammatory bowel disease (IBD) [6]. Moreover, it has been reported as a dynamic and sensitive biomarker of gut inflammation [33]. Besides, LCN2 also positively correlated with EED score. In addition, higher expression of LCN2 gene was found in duodenal biopsies of EED in transcriptomic study [34]. Another study also reported that there was increased expression of LCN2 gene in case of severe acute malnutrition (SAM) enteropathy [35]. Overall, our finding implies that LCN2 might be a potential biomarker for intestinal inflammation as well as it may be used as a predictor of EED.

In our analysis we also measured the level of plasma LRP1. Correlation among the plasma LRP1 and fecal biomarkers showed that LRP1 is negatively correlated with MPO, NEO, AAT and Reg1B. Moreover, LRP1 also negatively correlates with EED score. MPO, NEO, A1AT have been reported as biomarkers of intestinal inflammation and EED [26 29-31] and correlates negatively with LRP1. Therefore, these results support our supposition as we hypothesized that LRP1 may reduce intestinal inflammation. Overall, it can be inferred that LRP1 may contribute in the reduction of intestinal inflammation. Our finding is supported by another study, where it has been reported that LRP1 exerts anti-inflammatory effect upon LPS exposure [36]. LRP1 also functions in activation of lysosomal enzymes, phagocytosis as well as destruction of microorganisms thus LRP1 contributes in reduction of gut inflammation [16 19 37]. LRP1 was also found to be played a pivotal role in elimination of pathogen by scavenging membrane lipoproteins of microorganism during microbial invasion in another study [23]. On the other hand, intestinal inflammation has been observed in LRP1 knockout mice [4]. Altogether, these findings strongly suggest that LRP1 has an effect on the reduction of gut inflammation.

Confidential: For Review Only

Limitations and strengths

There were several limitations in our study. Firstly, we could not measure the genetic expression of LCN2 and LRP1 of participants due to invasive collection process of intestinal cell. Secondly, we did not examine the consequence of suppression of LRP1 expression of human to investigate whether decreased expression may or may not contribute to gut inflammation because of ethical consideration. Lack of age and sex matched healthy children was also a limitation in this study. However, this was the first study that investigated the relationship of LCN2 and LRP1 with biomarkers of EED.

CONCLUSIONS

Our findings suggest that LCN2 might be a potential biomarker to predict the gut inflammation and EED. However, increased level of LRP1 may contribute in alleviating intestinal inflammation that could be a potential therapeutic target to reduce the inflammation.

Acknowledgments We cordially thank all the participants and their mothers of the BEED study as well as the laboratory and field staffs at icddr,b for their contributions. icddr,b also gratefully acknowledges the donors (Bill & Melinda Gates Foundation) of this research protocol.

Contributors TA conceptualized the study. MMH wrote the manuscript and analyzed the data. MMH, MAG, FH did the laboratory analysis. MAG, SD, SMF critically reviewed the manuscript. MAA, MMH involved in statistical analysis. MM, TA involved in the development of study protocol. All authors commented on manuscript and approved the final version.

Funding This study was funded by the Bill & Melinda Gates Foundation. The investment ID was OPP1136751.(https://www.gatesfoundation.org/How-We-Work/Quick-Links/GrantsDatabase/Grants/2015/11/OPP1136751).

Competing interests The authors have no competing interest.

Patient consent for publication Written consent was collected from mother of the participants during enrollment.

Ethics approval The study protocol was approved by Institutional review board of the International Centre for Diarrhoeal Disease Research, Bangladesh (icddr,b). Protocol number-PR-16007.

Provenance and peer review Not commissioned; peer reviewed externally.

https://mc.manuscriptcentral.com/bmjpo

Confidential: For Review Only

Data availability statement The data set that was created during the study is not publicly available due to the restriction of funder. However, suggestion for data analysis can be made to corresponding author.

REFERENCES

1. Korpe PS, Petri Jr WA. Environmental enteropathy: critical implications of a poorly understood condition. *Trends in molecular medicine* 2012;18(6):328-36.
2. Lunn P, Northrop-Clewes C, Downes R. Intestinal permeability, mucosal injury, and growth faltering in Gambian infants. *The Lancet* 1991;338(8772):907-10.
3. Fahim SM, Das S, Sanin KI, et al. Association of Fecal Markers of Environmental Enteric Dysfunction with Zinc and Iron Status among Children at First Two Years of Life in Bangladesh. *The American journal of tropical medicine and hygiene* 2018;99(2):489-94.
4. Uchiyama R, Kupkova K, Shetty SJ, et al. Histone H3 lysine 4 methylation signature associated with human undernutrition. *Proceedings of the National Academy of Sciences* 2018;115(48):E11264-E73.
5. Flo TH, Smith KD, Sato S, et al. Lipocalin 2 mediates an innate immune response to bacterial infection by sequestrating iron. *Nature* 2004;432(7019):917-21.
6. Moschen AR, Adolph TE, Gerner RR, et al. Lipocalin-2: a master mediator of intestinal and metabolic inflammation. *Trends in Endocrinology & Metabolism* 2017;28(5):388-97.
7. Toyonaga T, Matsuura M, Mori K, et al. Lipocalin 2 prevents intestinal inflammation by enhancing phagocytic bacterial clearance in macrophages. *Scientific reports* 2016;6:35014.
8. Flower DR, North AC, Sansom CE. The lipocalin protein family: structural and sequence overview. *Biochimica et Biophysica Acta (BBA)-Protein Structure and Molecular Enzymology* 2000;1482(1-2):9-24.
9. Schaible UE, Kaufmann SH. Iron and microbial infection. *Nature Reviews Microbiology* 2004;2(12):946-53.
10. Goetz DH, Holmes MA, Borregaard N, et al. The neutrophil lipocalin NGAL is a bacteriostatic agent that interferes with siderophore-mediated iron acquisition. *Molecular cell* 2002;10(5):1033-43.
11. Chan YR, Liu JS, Pociask DA, et al. Lipocalin 2 is required for pulmonary host defense against Klebsiella infection. *The Journal of Immunology* 2009;182(8):4947-56.
12. Nairz M, Schroll A, Haschka D, et al. Lipocalin-2 ensures host defense against Salmonella Typhimurium by controlling macrophage iron homeostasis and immune response. *European journal of immunology* 2015;45(11):3073-86.
13. Wu H, Santoni-Rugiu E, Ralfkiaer E, et al. Lipocalin 2 is protective against E. coli pneumonia. *Respiratory research* 2010;11(1):96.
14. Yammine L, Zablocki A, Baron W, et al. Lipocalin-2 Regulates Epidermal Growth Factor Receptor Intracellular Trafficking. *Cell reports* 2019;29(7):2067-77. e6.
15. Ronco C, Legrand M, Goldstein SL, et al. Neutrophil gelatinase-associated lipocalin: ready for routine clinical use? An international perspective. *Blood purification* 2014;37(4):271-85.
16. Lillis AP, Van Duyn LB, Murphy-Ullrich JE, et al. LDL receptor-related protein 1: unique tissue-specific functions revealed by selective gene knockout studies. *Physiological reviews* 2008;88(3):887-918.
17. Gonias SL, Campana WM. LDL receptor–related protein-1: a regulator of inflammation in atherosclerosis, cancer, and injury to the nervous system. *The American journal of pathology* 2014;184(1):18-27.
18. Strickland DK, Gonias SL, Argraves WS. Diverse roles for the LDL receptor family. *Trends in Endocrinology & Metabolism* 2002;13(2):66-74.
19. Lim JJ, Grinstein S, Roth Z. Diversity and versatility of phagocytosis: roles in innate immunity, tissue remodeling, and homeostasis. *Frontiers in cellular and infection microbiology* 2017;7:191.
20. Lin L, Hu K. LRP-1: functions, signaling and implications in kidney and other diseases. *International journal of molecular sciences* 2014;15(12):22887-901.
21. Potere N, Del Buono MG, Mauro AG, et al. Low density lipoprotein receptor-related protein-1 in cardiac inflammation and infarct healing. *Frontiers in cardiovascular medicine* 2019;6:51.
22. Yancey PG, Blakemore J, Ding L, et al. Macrophage LRP-1 controls plaque cellularity by regulating efferocytosis and Akt activation. *Arteriosclerosis, thrombosis, and vascular biology* 2010;30(4):787-95.
23. May P. The low-density lipoprotein receptor-related protein 1 in inflammation. *Current opinion in lipidology* 2013;24(2):134-37.

Confidential: For Review Only

24. Mahfuz M, Das S, Mazumder RN, et al. Bangladesh environmental enteric dysfunction (BEED) study: protocol for a community-based intervention study to validate non-invasive biomarkers of environmental enteric dysfunction. *BMJ open* 2017;7(8):e017768.
25. Pradhan NA, Ali TS, Hasnani FB, et al. Measuring socio-economic status of an urban squatter settlement in Pakistan using WAMI Index. *J Pak Med Assoc* 2018;68:709-14.
26. Kosek M, Haque R, Lima A, et al. Fecal markers of intestinal inflammation and permeability associated with the subsequent acquisition of linear growth deficits in infants. *The American journal of tropical medicine and hygiene* 2013;88(2):390-96.
27. Prata MdMG, Havt A, Bolick D, et al. Comparisons between myeloperoxidase, lactoferrin, calprotectin and lipocalin-2, as fecal biomarkers of intestinal inflammation in malnourished children. *Journal of translational science* 2016;2(2):134.
28. Guerrant RL, Leite AM, Pinkerton R, et al. Biomarkers of environmental enteropathy, inflammation, stunting, and impaired growth in children in northeast Brazil. *PloS one* 2016;11(9):e0158772.
29. George CM, Oldja L, Biswas SK, et al. Fecal markers of environmental enteropathy are associated with animal exposure and caregiver hygiene in Bangladesh. *The American journal of tropical medicine and hygiene* 2015;93(2):269-75.
30. Arndt MB, Richardson BA, Ahmed T, et al. Fecal markers of environmental enteropathy and subsequent growth in Bangladeshi children. *The American journal of tropical medicine and hygiene* 2016;95(3):694-701.
31. George CM, Oldja L, Biswas S, et al. Geophagy is associated with environmental enteropathy and stunting in children in rural Bangladesh. *The American journal of tropical medicine and hygiene* 2015;92(6):1117-24.
32. Konikoff MR, Denson LA. Role of fecal calprotectin as a biomarker of intestinal inflammation in inflammatory bowel disease. *Inflammatory bowel diseases* 2006;12(6):524-34.
33. Chassaing B, Srinivasan G, Delgado MA, et al. Fecal lipocalin 2, a sensitive and broadly dynamic non-invasive biomarker for intestinal inflammation. *PloS one* 2012;7(9):e44328.
34. Kummerlowe C, Wallach T, Mwakamui S, et al. Single-cell profiling of environmental enteropathy reveals signatures of epithelial remodeling and immune activation in severe disease. *bioRxiv* 2021
35. Chama M, Amadi BC, Chandwe K, et al. Transcriptomic analysis of enteropathy in Zambian children with severe acute malnutrition. *EBioMedicine* 2019;45:456-63.
36. Luo L, Wall AA, Tong SJ, et al. TLR crosstalk activates LRP1 to recruit Rab8a and PI3Kγ for suppression of inflammatory responses. *Cell reports* 2018;24(11):3033-44.
37. Safina D, Schlitt F, Romeo R, et al. Low-density lipoprotein receptor-related protein 1 is a novel modulator of radial glia stem cell proliferation, survival, and differentiation. *Glia* 2016;64(8):1363-80.

Confidential: For Review Only

Exact p- value for "**Table 3** Correlation of LCN 2 and LRP1 with others fecal biomarkers and EED score"

A significant positive correlation was found among LCN2 and different biomarkers of intestinal inflammation like MPO (r = 0.19, **p = 0.005**), NEO (r = 0.20, **p = 0.004**), Calprotectin (r= 0.3, **p = 0.0001**), and Reg1B (r = 0.20, **p = 0.003**). LCN2 also positively correlated with EED score (r = 0.20, **p = 0.003**). On the other hand, LRP1 was negatively correlated with MPO (r = **-** 0.18, **p = 0.006**), NEO (r = -0.30, **p = 0.0001**), A1AT (r = - 0.18**, p = 0.006**), Reg1B (r= -0.2, **p = 0.003**) as well as EED score (r = -0.29, **p = 0.0001**).

Confidential: For Review Only

BMJ Paediatrics Open

Association of Lipocalin-2 and Low-density lipoprotein receptor related protein-1 (LRP1) with biomarkers of Environmental Enteric Dysfunction (EED) among under 2 children in Bangladesh: Results from a community based intervention study

Journal:	*BMJ Paediatrics Open*
Manuscript ID	bmjpo-2021-001138.R2
Article Type:	Original research
Date Submitted by the Author:	13-Jul-2021
Complete List of Authors:	Hasan, Md. Mehedi ; International Centre for Diarrhoeal Disease Research Bangladesh<br>Gazi, Md Amran; International Centre for Diarrhoeal Disease Research Bangladesh, NCSD<br>Das, Subhasish; International Centre for Diarrhoeal Disease Research Bangladesh<br>Fahim, Shah Mohammad ; International Centre for Diarrhoeal Disease Research Bangladesh<br>Hossaini, Farzana ; International Centre for Diarrhoeal Disease Research Bangladesh<br>Alam, Md. Ashraful ; International Centre for Diarrhoeal Disease Research Bangladesh,<br>Mahfuz, Mustafa; International Centre for Diarrhoeal Disease Research Bangladesh<br>Ahmed, Tahmeed; International Centre for Diarrhoeal Disease Research, Bangladesh (icddr,b), Clinical Sciences Division (CSD); Centre for Nutrition and Food Security (CNFS)
Keywords:	Biochemistry, Gastroenterology, Growth

SCHOLARONE™
Manuscripts

I, the Submitting Author has the right to grant and does grant on behalf of all authors of the Work (as defined in the below author licence), an exclusive licence and/or a non-exclusive licence for contributions from authors who are: i) UK Crown employees; ii) where BMJ has agreed a CC-BY licence shall apply, and/or iii) in accordance with the terms applicable for US Federal Government officers or employees acting as part of their official duties; on a worldwide, perpetual, irrevocable, royalty-free basis to BMJ Publishing Group Ltd ("BMJ") its licensees and where the relevant Journal is co-owned by BMJ to the co-owners of the Journal, to publish the Work in this journal and any other BMJ products and to exploit all rights, as set out in our licence.

The Submitting Author accepts and understands that any supply made under these terms is made by BMJ to the Submitting Author unless you are acting as an employee on behalf of your employer or a postgraduate student of an affiliated institution which is paying any applicable article publishing charge ("APC") for Open Access articles. Where the Submitting Author wishes to make the Work available on an Open Access basis (and intends to pay the relevant APC), the terms of reuse of such Open Access shall be governed by a Creative Commons licence – details of these licences and which Creative Commons licence will apply to this Work are set out in our licence referred to above.

Other than as permitted in any relevant BMJ Author's Self Archiving Policies, I confirm this Work has not been accepted for publication elsewhere, is not being considered for publication elsewhere and does not duplicate material already published. I confirm all authors consent to publication of this Work and authorise the granting of this licence.

Confidential: For Review Only

Association of Lipocalin-2 and Low-density lipoprotein receptor related protein-1 (LRP1) with biomarkers of Environmental Enteric Dysfunction (EED) among under 2 children in Bangladesh: Results from a community based intervention study

Md. Mehedi Hasan[1], Md. Amran Gazi[1]*, Subhasish Das[1], Shah Mohammad Fahim[1], Farzana Hossaini[1], Md. Ashraful Alam [1], Mustafa Mahfuz[1], Tahmeed Ahmed[1]

[1]Nutrition and Clinical Services Division, International Centre for Diarrheal Disease Research, Bangladesh (icddr,b), Dhaka, Bangladesh

Authors' addresses:

1. Md. Mehedi Hasan, MSc , Senior Research Officer, Nutrition and Clinical Services Division, icddr,b, Dhaka, Bangladesh, Email: md.hasan@icddrb.org
2. Md. Amran Gazi, MSc, Assistant Scientist, Nutrition and Clinical Services Division, icddr,b, Dhaka, Bangladesh, Email: amran.gazi@icddrb.org
3. Subhasish Das, MBBS, MPH, Assistant Scientist, Nutrition and Clinical Services Division, icddr,b, Dhaka, Bangladesh, Email: subhasish.das@icddrb.org
4. Shah Mohammad Fahim, MBBS, MPH; Research Investigator, Nutrition and Clinical Services Division, icddr,b, Dhaka, Bangladesh; Email: mohammad.fahim@icddrb.org
5. Farzana Hossaini, MSc, Senior Research Assistant, Nutrition and Clinical Services Division, icddr,b, Dhaka, Bangladesh, Email: farzanasacky@gmail.com
6. Md. Ashraful Alam, MSc, Assistant Scientist, Nutrition and Clinical Services Division, icddr,b, Dhaka, Bangladesh, Email: mashraful@icddrb.org
7. Mustafa Mahfuz, MBBS, MPH, Associate Scientist, Nutrition and Clinical Services Division, icddr,b, Dhaka, Bangladesh, Email: mustafa@icddrb.org
8. Tahmeed Ahmed, MBBS, PhD, Executive Director, icddr,b, Dhaka, Bangladesh, Email: tahmeed@icddrb.org

***Correspondence to: Md. Amran Gazi,** Nutrition and Clinical Services Division, International Centre for Diarrhoeal Disease Research (icddr,b), Dhaka-1212, Bangladesh, **Email:** amran.gazi@icddrb.org

Word count: Abstract: 235; Main text: 1729

Number of table: 3

Confidential: For Review Only

ABSTRACT

Background Environmental enteric dysfunction (EED) is thought to occur from persistent intestinal inflammation. Studies also revealed the association of Lipocalin-2 (LCN2) and Low-density lipoprotein receptor related protein-1 (LRP1) with intestinal inflammation. Therefore, we intended to explore the relationship of LCN2 and LRP1 with gut inflammation and biomarkers of EED in Bangladeshi malnourished children.

Methods A total of 222 children (Length-for-age z-score (LAZ) <−1) aged 12-18 months were enrolled in this study in a cross sectional manner. Among the participants, 115 were stunted (LAZ <-2) and 107 were at risk of being stunted (LAZ -1 to -2) children. Plasma and fecal biomarkers were measured using ELISA. Spearman's rank correlation was done to see the correlation among LCN2, LRP1 and biological biomarkers.

Results LCN2 correlates positively with Myeloperoxidase (r = 0.19, p = 0.005), Neopterin (r = 0.20, p = 0.004), Calprotectin (r= 0.3, p = 0.0001), Reg1B (r = 0.20, p = 0.003) and EED score (r = 0.20, p = 0.003). Whereas, LRP1 correlates negatively with Myeloperoxidase (r = - 0.18, p = 0.006), Neopterin (r = - 0.30, p = 0.0001), Alpha-1-antitrypsin (r = - 0.18, p = 0.006), Reg1B (r= -0.2, p = 0.003) and EED score (r = - 0.29, p = 0.0001).

Conclusions Our findings imply that LCN2 might be a promising biomarker to predict gut inflammation and EED. Whereas, increased level of LRP1 may contribute to alleviating intestinal inflammation.

Key words: Lipocalin-2, LRP1, gut inflammation, EED, Bangladesh

Confidential: For Review Only

What is known about the subject?

- Environmental enteric dysfunction (EED) is highly prevalent in low and middle income countries which affect approximately 40% of all children with age less than 5 years.
- EED causes linear growth faltering or stunting which has the manifestations including reduced neurodevelopmental and cognitive function and increased morbidity and mortality in childhood.
- The main challenge to tackle the EED is lack of non-invasive biomarker signature to diagnose the EED and take proper treatment initiative.

What this study add?

- There are significant positive correlation of Lipocalin-2 and significant negative correlation of LRP-1 with biomarkers of EED.
- Study findings strongly infer that LCN2 might be a promising biomarker to predict gut inflammation and EED
- Study findings also imply that LRP1 could be a therapeutic target to alleviate intestinal inflammation.

Confidential: For Review Only

INTRODUCTION

Environmental enteric dysfunction (EED) is a subacute intestinal inflammation highly prevalent in low and middle income countries which is defined by crypt hyperplasia, villous atrophy, reduction of mucus layer and infiltration of lymphocytes in lamina propria [1]. EED affects approximately 40% of all children with age less than 5 years in resource poor settings and causes linear growth faltering or stunting [2]. It is thought to occur from repeated enteric infections due to poor hygiene and unsanitary environmental conditions, which in turn results in intestinal inflammation, a major driver of occurring EED [3]. Studies have elicited the relation of Lipocalin-2 (LCN2) and low-density lipoprotein receptor-related protein-1(LRP1) with intestinal inflammation. LRP1 knockout has been found to be associated with increased intestinal inflammation in animal model [4], and LCN2-deficient mice are more prone to bacterial infection [5]. On the other hand, higher expression of LCN2 has been reported in various inflammatory conditions [6]. Hence, it is necessary to explore the relation of LCN2 and LRP1 with intestinal inflammation in the context of EED.

LCN2 is a bacteriostatic peptide, a member of lipocalin super family, known as neutrophil gelatinase B-associated lipocalin (NGAL) [7 8]. One of the most recognized functions of LCN2 is to prevent the bacterial uptake of iron crucial for their growth [9 10]. It has been reported that LCN2-deficient mice shows higher mortality rates following infection with *Escherichia coli* than wild type [5]. Study also reported that LCN2 shows preventive mechanism against intestinal inflammation through bacterial clearance by phagocytosis in macrophages [7]. Moreover, it exerts defensive mechanism against *Salmonella typhimurium, Klebsiella pneumoniae* and *E. coli* in different studies [11-13]. Besides the defensive mechanism, LCN2 has emerged as promising biomarker of inflammation. It has been reported as hallmark for the onset of kidney diseases as well as indicator of inflammation in various diseases [6 14 15]. However, there is no study that has conducted to investigate the level of LCN2 in Bangladeshi malnourished children. Therefore, the relation of LCN2 with intestinal inflammation and EED are yet to be elucidated.

LRP1 is an endocytic receptor that facilitates the binding and endocytic transportation of a broad range of biological ligands as well as various molecules function in immune system [16-18]. It has a vital role in phagocytosis which is an essential component of the innate immune response, crucial for removal of infectious agents [16 19]. Moreover, LRP1 promotes tissue-resident macrophage-survival by activating Akt pathway [20]. Macrophages show the initial defensive mechanism by eliminate noxious agents including bacteria, irritants and necrotic cells [21], whereas LRP1 deficiency causes increased macrophage apoptosis [22]. LRP1 also mediates

Confidential: For Review Only

pathogen elimination by scavenging membrane lipoproteins of microorganism during infection [23]. Therefore, it can be hypothesized that LRP1 may reduce the gut inflammation by showing defensive mechanism against microbial invasion. In this study, we intended to investigate the level of LCN2 and LRP1 in malnourished children of Bangladesh and their relations with fecal markers of intestinal inflammation and EED.

METHODS

Study participants, study site and ethical consideration

To perform the analysis for this study, data were taken from "Bangladesh Environmental Enteric Dysfunction (BEED)" study. The study protocol was published earlier [24]. It is a community based intervention study to validate noninvasive biomarkers of EED. A total of 222 participants were included in this analysis in a cross sectional manner, among them 115 were stunted (LAZ <-2) and 107 were at risk of being stunted (LAZ -1 to -2). Study participants were enrolled from Bauniabadh, a slum of Mirpur, Dhaka, Bangladesh. The study protocol (PR-16007) was approved by the institutional review board of International Centre for Diarrheal Disease Research, Bangladesh (icddr,b). Written consent was obtained from mother of the participant after explaining about the study.

Confidential: For Review Only

Data acquisition, sample collection and laboratory assay

Anthropometric and socio-economic data were collected from study participants by trained staff. Length-for age z-score (LAZ), Weight-for length z-score (WLZ), Weight-for age z-score (WAZ), Mid-upper arm circumference (MUAC) and Head circumference of the study participants were calculated as anthropometric indices. A total of 4 ml of blood was collected by medical doctor following all the aseptic conditions and transported to laboratory maintaining cold condition (-4° c). Plasma was collected by centrifugation of blood at 3000 xg for 10 minutes. Aliquots of biological samples were stored at -80°C until biomarker analyses. Plasma biomarkers including LRP1 (BIOMATIK, USA), sCD14 (R& D system, USA), C-reactive protein (Immundiagnostik, Germany), Alpha-1 acid glycoprotein (Alpco, NH), and ferritin (ORGENTEC, Germany) were measured by using available ELISA kits. Atomic absorption spectrometry was used to measure the level of Zinc in plasma. Stool sample were utilized to detect the level of biomarkers including LCN2 (R&D system, USA), Neopterin (GenWay Biotech, California), Myeloperoxidase (Alpco, New Hampshire), Alpha-1 antitrypsin (Biovendor, North Carolina), Reg1B (TechLab, USA), Calprotectin (BU¨HLMANN, Switzerland)

by using available ELISA kit. All the laboratory analyses were performed at the parasitology laboratory of icddr,b.

Statistical analyses

Data analyses were performed to present the characteristics of the study participants. Data were presented as median with inter-quartile ranges when variables show skewed distributions, while categorical variables were showed as frequencies with percentages. The variables showing normal distribution were presented as mean with standard deviation (SD). Mann-Whitney U test was used to detect the differences in the age, family income, plasma biomarkers including C-reactive protein (CRP), Alpha-1-acid glycoprotein (AGP), sCD14, LRP1, ferritin and zinc as well as fecal biomarkers including Myeloperoxidase (MPO), Neopterin (NEO), Alpha-1 antitrypsin (A1AT), Calprotectin, Reg1B and LCN2; whereas Chi-square test was performed for comparing categorical variables of the participants. Student's t-test was performed to see the differences in length, weight, MUAC, head circumference, LAZ, WLZ, WAZ, maternal height and WAMI index (Water, sanitation, hygiene, Asset, Maternal education and Income index, ranging from 0 to 1) [25]. Spearman's correlation coefficient (*r*) was calculated to see the correlation of LRP1 and LCN2 with others fecal biomarkers and EED score (ranging from 0 to 10) that has been calculated from MPO, NEO and A1AT using the method published earlier [26]. In this analysis, p-value <0.05 was considered statistically significant. All the analyses were performed using STATA version -13.

Patient and public involvement

No patient or public were involved with the research design, protocol development and participant's enrollment. None of them will be involved during dissemination of findings.

RESULTS

Anthropometric, socioeconomic and biochemical parameters of the study participants

Table-1 presents the anthropometric and socioeconomic characteristics of the participants. Among the participants nearly half were female.

https://mc.manuscriptcentral.com/bmjpo

Confidential: For Review Only

Table 1 Anthropometric and socioeconomic characteristics of the study participants

	Overall (n=222)
Child age (month), Median (Q1,Q2))	18.3 (16.5, 20.3)
Female sex, n (%)	116 (52.3)
Weight in kg, Mean (SD)	9.05 (0.96)
Length in cm, Mean (SD)	76.7 (2.85)
MUAC in cm, Mean (SD)	14.2 (0.93)
Head circumference in cm, Mean (SD)	44.7 (1.38)
Weight for age z score, Mean (SD)	-1.44 (0.86)
Length for age z score, Mean (SD)	-1.82 (0.79)
Weight for length z score, Mean (SD)	-0.774 (0.91)
Maternal height in cm, Mean (SD)	149 (5.09)
Family income per month (USD),Median (Q1,Q2)	179 (124, 239)
Improved sanitation, n (%)	152 (68.5)
WAMI†, Mean (SD)	0.598 (0.134)

† WAMI: Water, sanitation, hygiene, Asset, Maternal education and Income (ranging from 0 to 1)

Categorical variables (Sex and Improved sanitation)

The levels of plasma biomarkers including LRP1, AGP, CRP, sCD14, Ferritin and Zinc and fecal biomarkers including MPO, NEO, A1AT, Calprotectin, Reg1B and LCN2 were presented in Table-2.

https://mc.manuscriptcentral.com/bmjpo

Confidential: For Review Only

Table 2 Levels of plasma and fecal biomarkers of the study participants

	Overall (n=222)
Plasma biomarkers	
LRP1 (ng/ml), Median (Q1, Q3)	1080 (796, 1390)
AGP (mg/dl), Median (Q1, Q3)	91.9 (69.0, 118)
CRP (mg/L), Median (Q1, Q3)	0.820 (0.284, 2.84)
sCD14 (ng/ml), Median (Q1, Q3)	1760 (1470, 2120)
Ferritin (ng/ml), Median (Q1, Q3)	13.8 (7.17, 25.1)
Zinc (mg/L), Median (Q1, Q3)	0.760 (0.68, 0.84)
Fecal biomarkers	
MPO (ng/ml), Median (Q1, Q3)	1720 (796, 3720)
Neopterin (nmol/L), Median (Q1, Q3)	1240 (567, 2690)
A1AT (mg/g), Median (Q1, Q3)	0.294 (0.13, 0.52)
Calprotectin (µg/g), Median (Q1, Q3)	372 (208, 677)
Reg1B (µg/ml), Median (Q1, Q3)	48.8 (13.9, 88.4)
LCN 2 (µg/g), Median (Q1, Q3)	134 (41.6, 280)

Correlation of LCN 2 and LRP1 with biomarkers and EED score

A significant positive correlation was found among LCN2 and different biomarkers of intestinal inflammation like MPO, NEO, Calprotectin and Reg1B. LCN2 also positively correlated with EED score. However, LRP1 was negatively and significantly correlated with MPO, NEO, A1AT, Reg1B and EED score. Correlations among different variables are illustrated in table-3.

Table 3 **Correlation of LCN 2 and LRP1 with others fecal biomarkers and EED score

Variables	LRP1	LCN 2
LRP1	1.00	
LCN 2	-0.04	1.00
MPO	-0.18*	0.19*
NEO	-0.30*	0.20*
A1AT	-0.18*	-0.005
Calprotectin	-0.12	0.30*
Reg1B	-0.20*	0.20*
EED score	-0.29*	0.20*

**Statistically significant (Exact p-value are shown in supplementary file)*

https://mc.manuscriptcentral.com/bmjpo

Confidential: For Review Only

DISCUSSION

Our results reveal that LCN2 positively correlates with biomarkers of intestinal inflammation including MPO, NEO, Calprotectin and Reg1B. These results are in accordance with the findings of another study, where LCN2 has been found to be positively correlated with MPO and Calprotectin [27]. Another study also reported positive correlation between LCN2 and Reg1-B, this is also in line with our findings [28]. Moreover, MPO, NEO, Caprotectin have been emerged as a biomarkers of intestinal inflammation as well as predictor of EED [26 29-32]. Therefore, LCN2 might be a potential biomarker to diagnose intestinal inflammation and EED. Study also reported that LCN2 is released from various cell types and up-regulated in tissue damaging conditions such as ulcerative colitis, infection and burn injury [6 27]. It has been conceded as an promising biomarker of inflammation, infection, ischemia as well as kidney damage [6]. LCN2 is highly expressed in intestinal epithelial cell in case of inflammatory bowel disease (IBD) [6]. Moreover, it has been reported as a dynamic and sensitive biomarker of gut inflammation [33]. Besides, LCN2 also positively correlated with EED score. In addition, higher expression of LCN2 gene was found in duodenal biopsies of EED in transcriptomic study [34]. Another study also reported that there was increased expression of LCN2 gene in case of severe acute malnutrition (SAM) enteropathy [35]. Overall, our finding implies that LCN2 might be a potential biomarker for intestinal inflammation as well as it may be used as a predictor of EED.

In our analysis we also measured the level of plasma LRP1. Correlation among the plasma LRP1 and fecal biomarkers showed that LRP1 is negatively correlated with MPO, NEO, A1AT and Reg1B. Moreover, LRP1 also negatively correlates with EED score. MPO, NEO, A1AT have been reported as biomarkers of intestinal inflammation and EED [26 29-31] and correlates negatively with LRP1. Therefore, these results support our supposition as we hypothesized that LRP1 may reduce intestinal inflammation. Overall, it can be inferred that LRP1 may contribute in the reduction of intestinal inflammation. Our finding is supported by another study, where it has been reported that LRP1 exerts anti-inflammatory effect upon LPS exposure [36]. LRP1 also functions in activation of lysosomal enzymes, phagocytosis as well as destruction of microorganisms thus LRP1 contributes in reduction of gut inflammation [16 19 37]. LRP1 was also found to be played a pivotal role in elimination of pathogen by scavenging membrane lipoproteins of microorganism during microbial invasion in another study [23]. On the other hand, intestinal inflammation has been observed in LRP1 knockout mice [4]. Altogether, these findings strongly suggest that LRP1 has an effect on the reduction of gut inflammation.

Confidential: For Review Only

Limitations and strengths

There were several limitations in our study. Firstly, we could not measure the genetic expression of LCN2 and LRP1 of participants due to invasive collection process of intestinal cell. Secondly, we did not examine the consequence of suppression of LRP1 expression of human to investigate whether decreased expression may or may not contribute to gut inflammation because of ethical consideration. Lack of age and sex matched healthy children was also a limitation in this study. However, this was the first study that investigated the relationship of LCN2 and LRP1 with biomarkers of EED.

CONCLUSIONS

Our findings suggest that LCN2 might be a potential biomarker to predict the gut inflammation and EED. However, increased level of LRP1 may contribute in alleviating intestinal inflammation that could be a potential therapeutic target to reduce the inflammation.

Acknowledgments We cordially thank all the participants and their mothers of the BEED study as well as the laboratory and field staffs at icddr,b for their contributions. icddr,b also gratefully acknowledges the donors (Bill & Melinda Gates Foundation) of this research protocol.

Contributors TA conceptualized the study. MMH wrote the manuscript and analyzed the data. MMH, MAG, FH did the laboratory analysis. MAG, SD, SMF critically reviewed the manuscript. MAA, MMH involved in statistical analysis. MM, TA involved in the development of study protocol. All authors commented on manuscript and approved the final version.

Funding This study was funded by the Bill & Melinda Gates Foundation. The investment ID was OPP1136751.(https://www.gatesfoundation.org/How-We-Work/Quick-Links/GrantsDatabase/Grants/2015/11/OPP1136751).

Competing interests The authors have no competing interest.

Patient consent for publication Written consent was collected from mother of the participants during enrollment.

Ethics approval The study protocol was approved by Institutional review board of the International Centre for Diarrhoeal Disease Research, Bangladesh (icddr,b). Protocol number-PR-16007.

Provenance and peer review Not commissioned; peer reviewed externally.

Confidential: For Review Only

Data availability statement The data set that was created during the study is not publicly available due to the restriction of funder. However, suggestion for data analysis can be made to corresponding author.

REFERENCES

1. Korpe PS, Petri Jr WA. Environmental enteropathy: critical implications of a poorly understood condition. *Trends in molecular medicine* 2012;18(6):328-36.
2. Lunn P, Northrop-Clewes C, Downes R. Intestinal permeability, mucosal injury, and growth faltering in Gambian infants. *The Lancet* 1991;338(8772):907-10.
3. Fahim SM, Das S, Sanin KI, et al. Association of Fecal Markers of Environmental Enteric Dysfunction with Zinc and Iron Status among Children at First Two Years of Life in Bangladesh. *The American journal of tropical medicine and hygiene* 2018;99(2):489-94.
4. Uchiyama R, Kupkova K, Shetty SJ, et al. Histone H3 lysine 4 methylation signature associated with human undernutrition. *Proceedings of the National Academy of Sciences* 2018;115(48):E11264-E73.
5. Flo TH, Smith KD, Sato S, et al. Lipocalin 2 mediates an innate immune response to bacterial infection by sequestrating iron. *Nature* 2004;432(7019):917-21.
6. Moschen AR, Adolph TE, Gerner RR, et al. Lipocalin-2: a master mediator of intestinal and metabolic inflammation. *Trends in Endocrinology & Metabolism* 2017;28(5):388-97.
7. Toyonaga T, Matsuura M, Mori K, et al. Lipocalin 2 prevents intestinal inflammation by enhancing phagocytic bacterial clearance in macrophages. *Scientific reports* 2016;6:35014.
8. Flower DR, North AC, Sansom CE. The lipocalin protein family: structural and sequence overview. *Biochimica et Biophysica Acta (BBA)-Protein Structure and Molecular Enzymology* 2000;1482(1-2):9-24.
9. Schaible UE, Kaufmann SH. Iron and microbial infection. *Nature Reviews Microbiology* 2004;2(12):946-53.
10. Goetz DH, Holmes MA, Borregaard N, et al. The neutrophil lipocalin NGAL is a bacteriostatic agent that interferes with siderophore-mediated iron acquisition. *Molecular cell* 2002;10(5):1033-43.
11. Chan YR, Liu JS, Pociask DA, et al. Lipocalin 2 is required for pulmonary host defense against Klebsiella infection. *The Journal of Immunology* 2009;182(8):4947-56.
12. Nairz M, Schroll A, Haschka D, et al. Lipocalin-2 ensures host defense against Salmonella Typhimurium by controlling macrophage iron homeostasis and immune response. *European journal of immunology* 2015;45(11):3073-86.
13. Wu H, Santoni-Rugiu E, Ralfkiaer E, et al. Lipocalin 2 is protective against E. coli pneumonia. *Respiratory research* 2010;11(1):96.
14. Yammine L, Zablocki A, Baron W, et al. Lipocalin-2 Regulates Epidermal Growth Factor Receptor Intracellular Trafficking. *Cell reports* 2019;29(7):2067-77. e6.
15. Ronco C, Legrand M, Goldstein SL, et al. Neutrophil gelatinase-associated lipocalin: ready for routine clinical use? An international perspective. *Blood purification* 2014;37(4):271-85.
16. Lillis AP, Van Duyn LB, Murphy-Ullrich JE, et al. LDL receptor-related protein 1: unique tissue-specific functions revealed by selective gene knockout studies. *Physiological reviews* 2008;88(3):887-918.
17. Gonias SL, Campana WM. LDL receptor–related protein-1: a regulator of inflammation in atherosclerosis, cancer, and injury to the nervous system. *The American journal of pathology* 2014;184(1):18-27.
18. Strickland DK, Gonias SL, Argraves WS. Diverse roles for the LDL receptor family. *Trends in Endocrinology & Metabolism* 2002;13(2):66-74.
19. Lim JJ, Grinstein S, Roth Z. Diversity and versatility of phagocytosis: roles in innate immunity, tissue remodeling, and homeostasis. *Frontiers in cellular and infection microbiology* 2017;7:191.
20. Lin L, Hu K. LRP-1: functions, signaling and implications in kidney and other diseases. *International journal of molecular sciences* 2014;15(12):22887-901.
21. Potere N, Del Buono MG, Mauro AG, et al. Low density lipoprotein receptor-related protein-1 in cardiac inflammation and infarct healing. *Frontiers in cardiovascular medicine* 2019;6:51.
22. Yancey PG, Blakemore J, Ding L, et al. Macrophage LRP-1 controls plaque cellularity by regulating efferocytosis and Akt activation. *Arteriosclerosis, thrombosis, and vascular biology* 2010;30(4):787-95.
23. May P. The low-density lipoprotein receptor-related protein 1 in inflammation. *Current opinion in lipidology* 2013;24(2):134-37.

https://mc.manuscriptcentral.com/bmjpo

24. Mahfuz M, Das S, Mazumder RN, et al. Bangladesh environmental enteric dysfunction (BEED) study: protocol for a community-based intervention study to validate non-invasive biomarkers of environmental enteric dysfunction. *BMJ open* 2017;7(8):e017768.
25. Pradhan NA, Ali TS, Hasnani FB, et al. Measuring socio-economic status of an urban squatter settlement in Pakistan using WAMI Index. *J Pak Med Assoc* 2018;68:709-14.
26. Kosek M, Haque R, Lima A, et al. Fecal markers of intestinal inflammation and permeability associated with the subsequent acquisition of linear growth deficits in infants. *The American journal of tropical medicine and hygiene* 2013;88(2):390-96.
27. Prata MdMG, Havt A, Bolick D, et al. Comparisons between myeloperoxidase, lactoferrin, calprotectin and lipocalin-2, as fecal biomarkers of intestinal inflammation in malnourished children. *Journal of translational science* 2016;2(2):134.
28. Guerrant RL, Leite AM, Pinkerton R, et al. Biomarkers of environmental enteropathy, inflammation, stunting, and impaired growth in children in northeast Brazil. *PloS one* 2016;11(9):e0158772.
29. George CM, Oldja L, Biswas SK, et al. Fecal markers of environmental enteropathy are associated with animal exposure and caregiver hygiene in Bangladesh. *The American journal of tropical medicine and hygiene* 2015;93(2):269-75.
30. Arndt MB, Richardson BA, Ahmed T, et al. Fecal markers of environmental enteropathy and subsequent growth in Bangladeshi children. *The American journal of tropical medicine and hygiene* 2016;95(3):694-701.
31. George CM, Oldja L, Biswas S, et al. Geophagy is associated with environmental enteropathy and stunting in children in rural Bangladesh. *The American journal of tropical medicine and hygiene* 2015;92(6):1117-24.
32. Konikoff MR, Denson LA. Role of fecal calprotectin as a biomarker of intestinal inflammation in inflammatory bowel disease. *Inflammatory bowel diseases* 2006;12(6):524-34.
33. Chassaing B, Srinivasan G, Delgado MA, et al. Fecal lipocalin 2, a sensitive and broadly dynamic non-invasive biomarker for intestinal inflammation. *PloS one* 2012;7(9):e44328.
34. Kummerlowe C, Wallach T, Mwakamui S, et al. Single-cell profiling of environmental enteropathy reveals signatures of epithelial remodeling and immune activation in severe disease. *bioRxiv* 2021
35. Chama M, Amadi BC, Chandwe K, et al. Transcriptomic analysis of enteropathy in Zambian children with severe acute malnutrition. *EBioMedicine* 2019;45:456-63.
36. Luo L, Wall AA, Tong SJ, et al. TLR crosstalk activates LRP1 to recruit Rab8a and PI3Kγ for suppression of inflammatory responses. *Cell reports* 2018;24(11):3033-44.
37. Safina D, Schlitt F, Romeo R, et al. Low-density lipoprotein receptor-related protein 1 is a novel modulator of radial glia stem cell proliferation, survival, and differentiation. *Glia* 2016;64(8):1363-80.

Confidential: For Review Only

Exact p- value for "**Table 3** Correlation of LCN 2 and LRP1 with others fecal biomarkers and EED score"

A significant positive correlation was found among LCN2 and different biomarkers of intestinal inflammation like MPO (r = 0.19, **p = 0.005**), NEO (r = 0.20, **p = 0.004**), Calprotectin (r= 0.3, **p = 0.0001**), and Reg1B (r = 0.20, **p = 0.003**). LCN2 also positively correlated with EED score (r = 0.20, **p = 0.003**). On the other hand, LRP1 was negatively correlated with MPO (r = **-** 0.18, **p = 0.006**), NEO (r = -0.30, **p = 0.0001**), A1AT (r = - 0.18**, p = 0.006**), Reg1B (r= -0.2, **p = 0.003**) as well as EED score (r = -0.29, **p = 0.0001**).

Confidential: For Review Only
